# New insights into *Trypanosoma cruzi* genetic diversity, and its influence on parasite biology and clinical outcomes

**DOI:** 10.3389/fimmu.2024.1342431

**Published:** 2024-04-09

**Authors:** Marina Malheiros Araújo Silvestrini, Glaucia Diniz Alessio, Bruna Estefânia Diniz Frias, Policarpo Ademar Sales Júnior, Márcio Sobreira Silva Araújo, Carolina Malheiros Araújo Silvestrini, Gustavo Eustáquio Brito Alvim de Melo, Olindo Assis Martins-Filho, Andréa Teixeira-Carvalho, Helen Rodrigues Martins

**Affiliations:** ^1^ Integrated Biomarker Research Group, René Rachou Institute, Fiocruz Minas, Oswaldo Cruz Foundation, Belo Horizonte, Minas Gerais, Brazil; ^2^ Department of Pharmacy, Federal University of the Jequitinhonha and Mucuri Valleys, Diamantina, Minas Gerais, Brazil

**Keywords:** Chagas disease, *Trypanosoma cruzi*, DTU, infectivity, immune response, pathogenesis

## Abstract

Chagas disease, caused by *Trypanosoma cruzi*, remains a serious public health problem worldwide. The parasite was subdivided into six distinct genetic groups, called “discrete typing units” (DTUs), from TcI to TcVI. Several studies have indicated that the heterogeneity of *T. cruzi* species directly affects the diversity of clinical manifestations of Chagas disease, control, diagnosis performance, and susceptibility to treatment. Thus, this review aims to describe how *T. cruzi* genetic diversity influences the biology of the parasite and/or clinical parameters in humans. Regarding the geographic dispersion of *T. cruzi*, evident differences were observed in the distribution of DTUs in distinct areas. For example, TcII is the main DTU detected in Brazilian patients from the central and southeastern regions, where there are also registers of TcVI as a secondary *T. cruzi* DTU. An important aspect observed in previous studies is that the genetic variability of *T. cruzi* can impact parasite infectivity, reproduction, and differentiation in the vectors. It has been proposed that *T. cruzi* DTU influences the host immune response and affects disease progression. Genetic aspects of the parasite play an important role in determining which host tissues will be infected, thus heavily influencing Chagas disease’s pathogenesis. Several teams have investigated the correlation between *T. cruzi* DTU and the reactivation of Chagas disease. In agreement with these data, it is reasonable to suppose that the immunological condition of the patient, whether or not associated with the reactivation of the *T. cruzi* infection and the parasite strain, may have an important role in the pathogenesis of Chagas disease. In this context, understanding the genetics of *T. cruzi* and its biological and clinical implications will provide new knowledge that may contribute to additional strategies in the diagnosis and clinical outcome follow-up of patients with Chagas disease, in addition to the reactivation of immunocompromised patients infected with *T. cruzi*.

## 1 Introduction

Chagas disease, caused by the protozoan *Trypanosoma cruzi*, was discovered over a century ago and remains a serious public health concern worldwide (1). According to the World Health Organization (WHO), the disease affects approximately 6–7 million people, mainly in Latin America, with 75 million people at risk of infection and more than 10,000 deaths per year (2, 3).

*T. cruzi* is a flagellate digenetic protozoan belonging to the order Kinetoplastida and family Trypanosomatidae (1). This species is heterogeneous and consists of several subpopulations that circulate in diverse environments, presenting great genetic diversity (4, 5). The analysis of a large panel of clones derived from wild-type isolates confirmed that the genome size can vary up to 48% between *T. cruzi* strains, and this difference is significant for populations of the same species (6). Hybridization events have contributed to current *T. cruzi* population structures and distinct genetic subgroups’ evolution of the parasite (4, 7).

These *T. cruzi* subgroups have been studied with different molecular tools over the years and have received distinct designations, including zymodemes (8–10), schizodemes (11), biodemes (12, 13), clones (14), groups (15), lineages (16), clades (17), and discrete typing units (DTUs) (18). In a Satellite Meeting in 1999, the standardization of *T. cruzi* strain nomenclature was discussed to adopt a standard classification that taxonomists and the general community of researchers would use. An expert committee reviewed the subdivision of the *T. cruzi* strains considering biological, biochemical, and molecular parameters to classify the species into two principal groups: *T. cruzi* I and *T. cruzi* II. The apparent hybrid strain was named *T. cruzi* (19).

However, with the advances in molecular biology and knowledge of parasite biology, this classification showed limitations due to the great dispersion of characteristics between members of the same *T. cruzi* genetic group (20). In the last decades, several molecular approaches have been used to elucidate the population structure of *T. cruzi* and have revealed a variable number of relevant subgroups in *T. cruzi* species (21–27). These data suggest that investigations employing the smaller *T. cruzi* subdivisions present more specific correlations between genetic parasite and biological, clinical, and epidemiological properties of the Chagas disease (**Table 1**). Consequently, 10 years after the Satellite Meeting, the scientific community has advanced knowledge of the diversity of *T. cruzi*. Thus, the current classification consensus proposes six genetic groups or DTUs, referred to as TcI to TcVI (38), related to previous classifications according to different markers. A DTU restricted to bats (Tcbat) was also considered (5). Studies based on multiple molecular markers support the definitive classification of Tcbat as a seventh DTU closely related to TcI, mainly found in bats (39, 40) and registered in Colombia, Panama, Chile, Equator, and Brazil (36, 41, 42). TcI and TcII represent ancestral *T. cruzi* lineages, which, from hybridization events, give rise to TcIII and TcIV, respectively (43, 44). In contrast, Freitas et al. (2006) (26) suggested the existence of three ancestral *T. cruzi* lineages (TcI, TcII, and TcIII). Nonetheless, genetic analyses showed TcI and TcII traits in TcIV and TcIII isolates, supporting the first model (7, 43, 45). TcV and TcVI are considered genetic hybrid groups formed from TcII and TcIII, which are widely circulated in the domestic cycle in South American countries (46–49). However, more recently, they have been found to infect dogs in the United States (50).

**Table 1:** Association between *Trypanosoma cruzi* DTUs, related clinical form, and immune response.

DTU/Genetic group	Transmission	Related clinical form	Immune response	References
TcI/Colombian	Sylvatic and domestic	Cardiomyopathy and meningoencephalitis (less pathogenic with less parasitemia)	TNF, IL-2, IL-4, IL-6, IL-10, and IFN-γ in human cardiac cells	(28–31)
TcII/Y	Domestic	Mega-visceral syndrome (megacolon and megaesophagus) and fewer cases of cardiomyopathy	Higher levels of IL-1β, IL-2, IL-4, IL-6, IL-5, IL-17A, IL-18, and IL-13 in human sera. It has many virulence factors like enzymes and receptors to avoid the host immune response.	(5, 31–33)
TcIII/QMM3	Sylvatic	Cardiomyopathy and mega-visceral syndrome, fewer human cases	It is considered rare in human infection and needs more investigation on the immune response profile.	(5, 31)
TcIV/4167	Sylvatic	Cardiomyopathy and mega-visceral syndrome, fewer human cases	*In vitro* antigenic stimulation in monkeys showed higher levels of TNF and IFN-γ and lower production of IL-10.	(32, 34)
TcV/SO3	Domestic	Megavisceral syndrome and cardiomyopathy	In less aggressive forms of cardiomyopathy, it induced higher levels of IL-1 in the culture of peripheral blood mononuclear cells (PBMC) from Chagas disease patients.	(32, 35)
TcVI/CL	Domestic	Megavisceral syndrome and cardiomyopathy	In less aggressive forms of cardiomyopathy, it induced higher levels of IL-17 in the culture of peripheral blood mononuclear cells (PBMC) from Chagas disease patients.	(5, 35)
TcBat	Sylvatic	Anthropogenic deaths have been reported in countries such as Colombia and Brazil; the zoonotic potential of this genotype is being considered.	This DTU needs further investigation, including recent cases and the immune response.	(36)

Taken and adapted from Jiménez et al. (37).

Several studies have shown that the heterogeneity of *T. cruzi* species directly affects the diversity of clinical manifestations, the control, the laboratorial diagnosis performance, and the susceptibility to treatment in the Chagas disease (**Table 1**). Thus, this review aims to describe how *T. cruzi* genetic diversity influences the biology of the parasite and/or clinical parameters in humans.

## 2 Epidemiology and DTU geographic distribution

Chagas disease is endemic to 21 countries and is considered a neglected disease by the WHO (51–53). In some regions, especially in countries in the Southern Cone of America, the acute phase of the disease had a clear reduction due to the success of vector and blood bank control. Owing to the migration of individuals with Chagas disease and the possibility of transmission by other routes, such as blood transfusion, congenital, and organ transplants in individuals chronically infected with *T. cruzi* (54, 55), the infection has also acquired importance in non-endemic areas of Chagas disease (56, 57).

An outstanding characteristic of *T. cruzi* is its significant plasticity, characterized by genetic variability and intense intraspecific phenotypic diversity. Regarding geographic dispersion, evident differences in *T. cruzi* DTU distribution were observed in distinct areas. For example, all DTUs were identified in South America, while TcI, TcII, and TcIV were identified in North America, and only TcI and TcIV were detected in the hosts and vectors from Central America (42, 49). TcI is the most ubiquitous DTU found in the wild cycle throughout all endemic areas. In the domestic cycle of the *T. cruzi* infection, it was widely prevalent in patients from North and Central America, Colombia, and Venezuela (7, 42, 58–60); some cases have also been identified in Chile and Argentina (21, 42, 61). The TcI has been frequently associated with outbreaks of oral transmission in the Northern Amazon (39, 62), but not in other South American regions, where TcII, TcV, and TcVI are more related to chronic infection, regardless of the clinical manifestation of Chagas disease (63–65).

TcII is the main DTU detected in Brazilian patients from the Central and Southeastern regions, where there were also registers of TcVI as a secondary DTU (66, 67). TcII is abundant in Chile and Colombia and less frequent in other regions (21, 42, 68).

TcIII and TcVI were predominantly associated with sylvatic cycles of the *T. cruzi* infection. TcIII was detected in sylvatic hosts from Northeastern Venezuela to Argentina, but infection in dogs and humans has been reported in Colombia (69, 70) and Argentinean Chaco (71–73). TcIV is a relevant agent in Chagas disease in Venezuela (58). It has also been reported in oral outbreaks in the Brazilian Amazon region (39, 62, 74).

TcV and TcVI were observed predominantly in the Southern Cone greater Gran Chaco (Peru, Bolivia, Northern Chile, and Argentina), Paraguay, and Uruguay. There are some registers of TcV in the extreme South of Brazil and Paraguay (5, 73). Some cases of human *T. cruzi* infection with TcV and/or TcVI have been reported further north in Ecuador (75) and Colombia (41, 76). In Bolivia, all DTUs were identified as circulating in humans except TcIII (42), with a predominance of TcI and TcV.

In addition, to the ecogeographic diversity, the literature demonstrated variations in biological and biochemical characteristics between *T. cruzi* subpopulations. In 1909, Chagas already described the morphological differences in the blood forms of the parasite. Generally, the stout forms of *T. cruzi* remain in the bloodstream and are unable to invade host cells rapidly, while the slender forms invade cells and start the intracellular cycle more quickly. The variation in parasitemia and tissue tropism correlates with the morphology of the *T. cruzi* forms because different strains have variable numbers of each form (77, 78). Miles et al. (1981) (58) described the first evidence that chronic Chagas disease manifestation could differ according to strain type and geographical location. In Venezuela, patients rarely develop mega-syndromes, while central and eastern Brazil have common cardiac and mega-syndrome manifestations (58). Although considerable advances have been made in the understanding of the physiopathogenesis of Chagas disease, no prognostic factors still allow a conclusive definition of patients infected with *T. cruzi* who develop symptomatic clinical forms. This multifactorial process involves host, parasite and environmental features, so a broad approach must be considered to better understand the factors that influence the physiopathogenesis of Chagas disease (79–81). Considering the *T. cruzi* genetic diversity, the current data corroborate Miles’ observations since the digestive forms are associated with infections where TcII, TcV, and TcIV DTUs were identified, while indeterminate forms and cardiac forms could be related to different DTUs with greater or lesser severity depending on the geographic regions (73, 82, 83). Differences in the capacity of *T. cruzi* for cell penetration, infectivity, differential induction of the immune response, tissue tropism, and response to treatment have already been demonstrated (84–86), which could confer differential capacity for adaptation to the host, influencing the transmission, response to the treatment, and the course of the infection (5, 87). Several studies have also demonstrated that distinct *T. cruzi* strains could trigger different immune responses and be influenced by geographical location (31, 88–93).

In addition, *T. cruzi* mixed infections and reinfections can alter the natural course of infection compared to individual populations, giving the parasite new properties and hampering correlations (13, 89, 94–96).

## 3 Infectivity and virulence of *T. cruzi* DTUs

The first criteria for subdivision of *T. cruzi* were based on parameters such as infectivity and virulence in mice infected with distinct parasite strains (12, 97). Andrade and Magalhães (1997) (97) characterized Type I as strains with a prevalence of thin forms and macrophage tropism that multiply quickly, developing high parasitemia and consequently leading to the death of Swiss mice in the first days after infections (7th–12th days). Type II *T. cruzi* strains have a frequency of wide forms and myocardial tropism that multiply slowly and present irregular peaks of parasitemia between the 12th and 20th day after infection. Moreover, the authors considered Type III as a *T. cruzi* strain with a prevalence of wide forms and skeletal musculature tropism, which multiply slowly and present late parasitemia peaks (20th–30th days), as well as low mortality rates in Swiss mice.

The correlation between the genetic variability of *T. cruzi* and its biological properties (60) has been scientifically sustained and corroborated in several studies with strains and clonal stocks of the parasite *in vitro* (98–100), experimental mouse infections (84, 100–102), and vectors (103–106). In general, these studies demonstrated that TcI presented higher multiplication and survival in acellular and cellular cultures than TcII when the biological behavior of clones (13, 98, 99) and strains of *T. cruzi* was evaluated (97, 107, 108). Revollo et al. (1998) (99) and Nogueira-Paiva et al. (2015) (108) used the “Vero” cells (cells isolated from African Green monkey kidney obtained from the Laboratory of Parasitology at the Pasteur Institute in Paris) to show differences in multiplication and survival when comparing the growth of different DTUs of *T. cruzi in vitro*, whereas Andrade and Magalhães (1997) (97), Rimoldi et al. (2012) (107) and Andrade et al. (2010) (13) used different mice organs and cells to demonstrate the distinct biological behavior of *T. cruzi* genotypes. Oliveira et al. (2017) (100) showed different results regarding the biological behavior *in vitro* of parasites isolated from patients with chronic Chagas disease. These authors verified that one sample of TcII demonstrated more significant growth in acellular culture than TcI and TcVI, and two other samples of TcII showed similar growth. The authors also revealed that the infectivity of “Vero” cells after 72 h was similar for all DTUs. However, one sample of TcII DTU yielded a higher number of amastigotes per cell.

The genetic variability of *T. cruzi* can influence parasite infectivity, reproduction, and differentiation in vectors (103, 104, 109, 110). Previous studies have demonstrated that clones of TcI are better able to infect and complete the life cycle in the *Triatoma infestans* vector than TcII clones, indicating that the DTUs do not exhibit the same vectorial transmissibility (103, 104). De Abreu et al. (2022) (111) showed differences in vector susceptibility to infection and competence when two vector species were infected with TcI, TcII, and TcIV strains from Amazon (AM) and Paraná (PR) in single and mixed *T. cruzi* infections. It is verified that *Rhodnius robustus* showed vector competence for TcIV (AM) and TcI (AM) + TcIV (AM), and *Rhodnius pictipes* showed vector competence for TcI (AM) + TcIV (AM) and TcI (AM) + TcII (PR).

The biological behavior of *T. cruzi* DTUs such as parasitological parameters, infectivity, and virulence was also assayed in the vertebrate hosts (84, 90, 91, 97, 100, 112–121). Lana et al. (2000) (113) revealed that 19/20 (TcI), 32 (TcII), and 39 (TcV) clonal *T. cruzi* genotypes exhibited high, intermediate, and low maximum parasitemia peaks, respectively, indicating that the clonal genotypes differed significantly in their infectivity in BALB/c mice. Toledo et al. (2002) (84) verified the biological behavior of a broader panel of *T. cruzi* clonal stocks. They demonstrated differences in parasitemia, infectivity, mortality, tissue parasitism, and inflammatory processes during the acute and chronic phases of experimental *T. cruzi* infection in BALB/c mice. The authors concluded that closely related clonal *T. cruzi* genotypes, 19 vs. 20 (TcI) and 32 (TcII) vs. 39 (TcV), showed, in general, fewer differences among themselves than distantly related groups, 19 or 20 (TcI) vs. 32 (TcII) or 39 (TcV). Some studies have shown that TcI has a high ability to infect experimental BALB/c (85) and Swiss mice (97, 112), developing larger parasitemia than other *T. cruzi* genetic groups (85, 97, 112). However, it has been demonstrated that samples of TcI isolated from patients with chronic Chagas disease may also present low levels of parasitemia in Swiss mice (100, 118). Sales-Campos et al. (2015) (121) verified that strains belonging to TcI exhibited low levels of parasitemia in BALB/c mice. *T. cruzi* stocks belonging to TcI and TcIV from the Brazilian Amazon diverged in terms of biological and medical properties in Swiss mice, while TcIV displayed higher virulence and parasitemia parameter values, and TcI determined the most severe inflammatory process and higher frequency of tissue parasitism (117). These infectivity and virulence alterations in TcI may be associated with intra-DTU differences. In contrast, TcII isolated from *T. cruzi*-infected humans exhibits high infectivity and virulence in experimental Swiss mice (90, 100, 114, 116). Lisboa et al. (2007) (122) studied sylvatic *T. cruzi* stocks and verified higher parasitemia in Swiss mice infected with TcII than with TcI. Significant biological heterogeneity was also observed in *T. cruzi* TcII isolates derived from *L. rosalia* from the Atlantic Forest, unlike the TcII isolates derived from marsupials, which present similar profiles. Together, these data confirm the high level of intraspecific divergence in *T. cruzi* and that the behavior of isolates can vary according to geographic and host origin (85, 117, 122, 123).

Standard *T. cruzi* strains, representative of the major genotypes of the parasite, have also been used to evaluate the biological behavior of DTUs in experimental animals (91, 124). Magalhães et al. (2015) (119) showed that Colombiana (TcI) and Y (TcII) strains lead to the differential activation of human monocytes and T cells, which might influence disease progression. Corroborating this work, Duz et al. (2014) (91) indicated that dogs infected with the Colombiana strain (TcI) reached the parasitemia peak later than animals infected with the Y strain (TcII) and that the Y strain (TcII) triggered a more intense immune response during the acute phase of infection in dogs. This is consistent with the Colombiana strain (TcI) in experimental models that induced a milder infection than the Y strain (TcII).

Studies using other strains isolated from *T. cruzi* DTUs, such as TcIII, TcV, and TcVI, have also verified the infectivity and virulence of these genetic groups in C57BL/6J mice (115, 120). Ragone et al. (2015) (120) showed that TcVI induces high parasitemia, severe histological damage in the heart, and moderate lesions in C57BL/6J mice skeletal muscle. On the other hand, TcIII presents less parasitemia than TcVI and mild-to-moderate histological damage in the heart and skeletal muscle. Lastly, TcV induced subpatent parasitemia and mild lesions in the analyzed tissues.

In natural infections with *T. cruzi*, it is crucial to consider that mixed infections with multiple DTUs are common and can lead to changes in the biological properties of the parasite as well as the clinical course of Chagas disease (94, 95, 105, 113, 125–127). Some studies have demonstrated that *T. cruzi* clones with less ability to develop in invertebrate and vertebrate hosts during monoclonal infections can multiply in mixed infections (94, 104, 105, 113, 128). It has been demonstrated *in vivo* that the tissue tropism of one *T. cruzi* DTU could change in the presence of other DTUs (124, 129). Moreover, the histopathological damage and intensity of the inflammatory process resulting from these mixed *T. cruzi* infections also present remarkable variations (89, 130).

## 4 Immune response and cell interaction

The immune response against Chagas disease has been investigated for many years and can generate protective or pathogenic events. The host’s genetic background, in addition to the specific characteristics of the parasite, seems to play a relevant role in influencing the outcome of the disease (131). The murine model of Chagas disease has been well established and effectively reflects the key features of human *T. cruzi* infection. Notably, mice’s immune response and cytokine expression levels during the acute and chronic phases closely resemble those observed in human Chagas disease (132). Ferreira et al. (2018) (132) assessed aspects of the acute and chronic phases of *T. cruzi* infection using G (TcI) and CL (TcVI) strains in two distinct mouse lineages (C57BL/6 and BALB/c). These findings revealed that the CL strain established infection more rapidly than the G strain. Concurrently, the immune response in BALB/c mice was initiated earlier than that in C57BL/6 mice. In the acute phase, all animals responded to *T. cruzi* infection by elevated serum concentrations of cytokines, with BALB/c mice demonstrating a more regulated immune response than C57BL/6 mice. In the chronic phase, C57BL/6 mice continued to exhibit exacerbated cytokine and chemokine responses. Despite differences in *T. cruzi* tropism among parasite strains, host background can significantly influence immune responses throughout infection (132).

Previous studies have demonstrated variations in the features of *T. cruzi* infection based on the mouse lineage and parasite strain used in the infection (133–135). In a study conducted by Sanoja et al. (2013) (134), a comparative analysis of CD4+ T-cell subset dynamics was performed after infection with the Y strain during the acute and chronic phases in BALB/c and C57BL/6 mice. The findings revealed that C57BL/6 mice exhibited heightened levels of CD4+ T-cell infiltration and expression of Th1 cytokines in the heart, coupled with the presence of Treg cells. In contrast, BALB/c mice showed a higher heart parasite burden, lower heart CD4+ T-cell infiltration, and reduced levels of Th1 and inflammatory cytokines, but with an increased presence of Th17 cells. In the chronic phase, BALB/c mice continued to exhibit higher parasite burdens than C57BL/6 mice, along with elevated levels of interferon (IFN)-γ, tumor necrosis factor (TNF), interleukin (IL)-10, and transforming growth factor (TGF)-β (134). Domingues et al. (2020) (135) also explored the influence of host genetics on histopathological and immunological aspects following infection with the SC2005 *T. cruzi* strain (isolated from a patient with Chagas disease) in BALB/c and A mice. They observed that BALB/c mice displayed higher parasitemia and mortality rates than A mice. Despite both lineages demonstrating a resistant immune profile, characterized by an increase in CD8+ T cells in the heart, liver, and blood; an increase in CD19+ B cells in the liver; and high levels of proinflammatory cytokines, the response to infection occurred later in BALB/c mice. Consequently, A mice were less susceptible to *T. cruzi* infection. The host genetic background can influence the early development of a cytotoxic cellular response profile, crucial in developing a less severe manifestation of Chagas disease (135). Other studies also support the significance of host genetic background in the clinical manifestations of chronic Chagas disease (136, 137). Frade et al. (2013) (136) analyzed CCR5, CCL2, and MAL/TIRAP genes in patients with chronic chagasic cardiomyopathy. Their findings demonstrated that the CCL2rs2530797A/A and TIRAPrs8177376A/A genotypes were associated with increased susceptibility, whereas the CCR5rs3176763C/C genotype was associated with cardiomyopathy protection (136).

The entry of *T. cruzi* into cells is a multifactorial process (138). Recent studies have indicated that many molecules interacting with specific cell receptors are expressed on the surface of *T. cruzi*, allowing the invasion of several cell types and the modulation of cell metabolism to enable its survival (139). Among the proteins involved in this process are GPI-anchored mucins, glycophosphatidylinositol, transialidase, and cruzipain, which are involved in the differential capacity of cell adhesion of *T. cruzi* strains and evasion of the immune response (140, 141).

The expression levels and glycosylation pattern diversity of mucin-associated surface proteins (MASPs) can vary among different *T. cruzi* strains, influencing the parasite’s virulence and immune response. MASP is the second-largest multigene family in *T. cruzi* and comprises approximately 1,300 genes. MASP proteins are characterized by a highly variable and repetitive central region comprising peptides shared among all MASP members. The pronounced polymorphism within this family and its surface location on infective forms of *T. cruzi* imply that MASP actively participates in host–parasite interaction mechanisms. Initial proteomic findings related to the MASP family suggested potentially low expression levels, with only four proteins identified in the trypomastigote stage and one in the epimastigote stage. One hypothesis posits that MASPs may be predominantly expressed during the intermediate stages of the parasite’s life cycle or undergo post-translational modifications, such as distinct N-glycosylation (142). Subsequent research on these genes has revealed protein expression in epimastigotes, trypomastigotes, and amastigotes of *T. cruzi*. Moreover, MASPs could be secreted during the early stages of amastigote genesis *in vitro* at pH 5.0 (143, 144). The expression profiles of these proteins may vary throughout infection, with different strains exhibiting differential expression of various MASP family members (145). Notably, immature MASP proteins and their C-terminal portion have been identified in trypomastigotes’ exosomes, showing immunogenic properties (146, 147). Recent studies have demonstrated that host antibodies against MASPs may exhibit strain-specific recognition patterns. Understanding the variation in surface-expressed molecules and diversity of MASPs among different *T. cruzi* strains is crucial for unraveling the complexity of Chagas disease and developing targeted interventions, including vaccines and treatments. A few years ago, a synthetic peptide derived from *T. cruzi* MASP was tested as a vaccine candidate against Chagas disease (148). This vaccine candidate induced an 86% survival rate in the immunized group after challenge with a highly lethal dose of trypomastigotes. Immunized animals also exhibited the lowest parasite load in the heart, liver, and spleen compared with control animals, as demonstrated by real-time polymerase chain reaction (PCR) (148). Ongoing research endeavors continue to unveil the intricacies of *T. cruzi* diversity and its implications in disease pathogenesis (148).

Gp90 has been described as a molecule with an antiphagocytic effect in mammalian stages of *T. cruzi* (149) and is considered a negative modulator of infection (150). Gp82 and gp35/50 are also implicated in cell invasion and are expressed on the surface of metacyclic trypomastigotes of *T. cruzi*. The mucin-like gp35/50 is resistant to protease digestion and responsible for protecting the parasite from destruction by the oral route (150). Gp83 is a ligand employed by the parasite to attach to and enter phagocytic and non-phagocytic cells (140, 151). All were expressed on the surface of metacyclic trypomastigotes.

**Figure 1:**
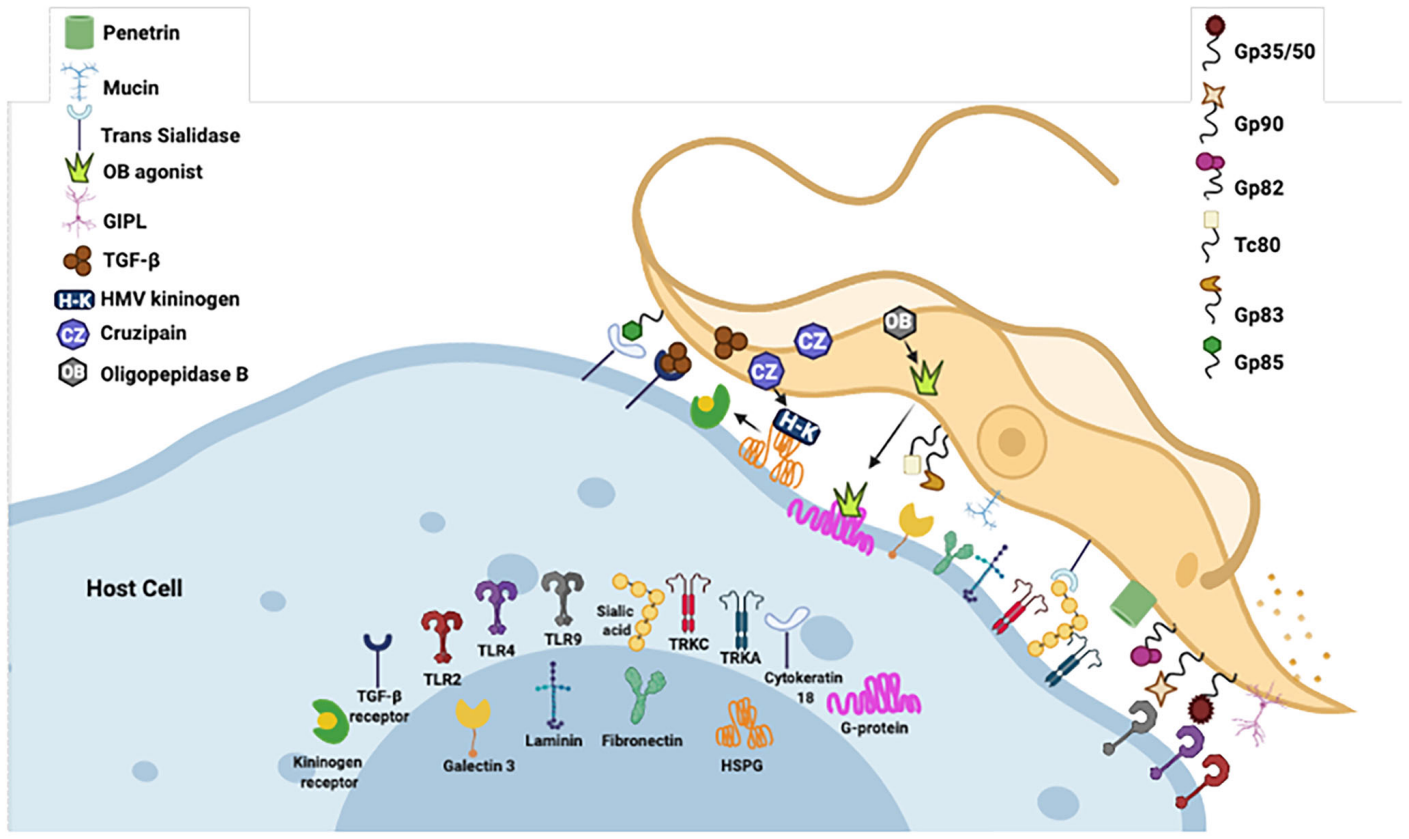
Examples of molecules expressed on the surface of *T. cruzi* that interact with specific cell receptors. Gp, glycoprotein; TLR, Toll-type receptors; MVs, microvesicles; LPS, lipopolysaccharide; PAMP, pathogen-associated molecular patterns; ssDNA, single-stranded DNA. Figure created by the author on the biorender.com platform, inspired by an Oswaldo Cruz Foundation website publication.

*T. cruzi* has been reported to activate Toll-like receptor 2 (TLR2), TLR4, and TLR9 (152, 153), and this innate recognition mechanism can influence cell activation and infectivity (**Figure 1**). TLRs are crucial components of the innate immune system, recognizing various pathogen-associated molecular patterns (PAMPs) and initiating immune responses (152–156). These receptors are important transmembrane proteins that confer a certain degree of specificity to the innate immune system cells. This innate recognition mechanism can influence cell activation and infectivity (**Figure 1**). Each TLR initiates downstream signaling that culminates in activating signaling pathways that regulate the expression of cytokines, chemokines, and interferons (153). Cell surface TLRs mainly recognize microbial membrane components such as lipids, lipoproteins, and proteins; intracellular TLRs recognize nucleic acids derived from bacteria and viruses and self-nucleic acids in disease conditions such as autoimmunity (155). TLR2, TLR4, and TLR9 are specifically mentioned in the context of *T. cruzi* infection. TLR2 recognizes various PAMPs, including lipoproteins and glycolipids (152–154, 156, 157). Studies have shown that TLR2 can be activated by parasite-derived molecules, contributing to the host immune response (153). TLR4 is primarily associated with bacterial infections for recognizing lipopolysaccharide (LPS), and some studies suggest its involvement in parasitic infections as well. The activation may occur through the recognition of parasite components (153). TLR9 recognizes bacterial and viral DNA that is rich in unmethylated CpG-DNA motifs; in the case of *T. cruzi*, the activation may be related to the presence of parasitic DNA, and additional studies attributed the production of proinflammatory cytokines (TNF and IL-12) to the cooperative activation of TLR2 and TLR9 (152, 157). This recognition can lead to the induction of pro-inflammatory responses and the initiation of an effective immune reaction against the parasite.

**Figure 2:**
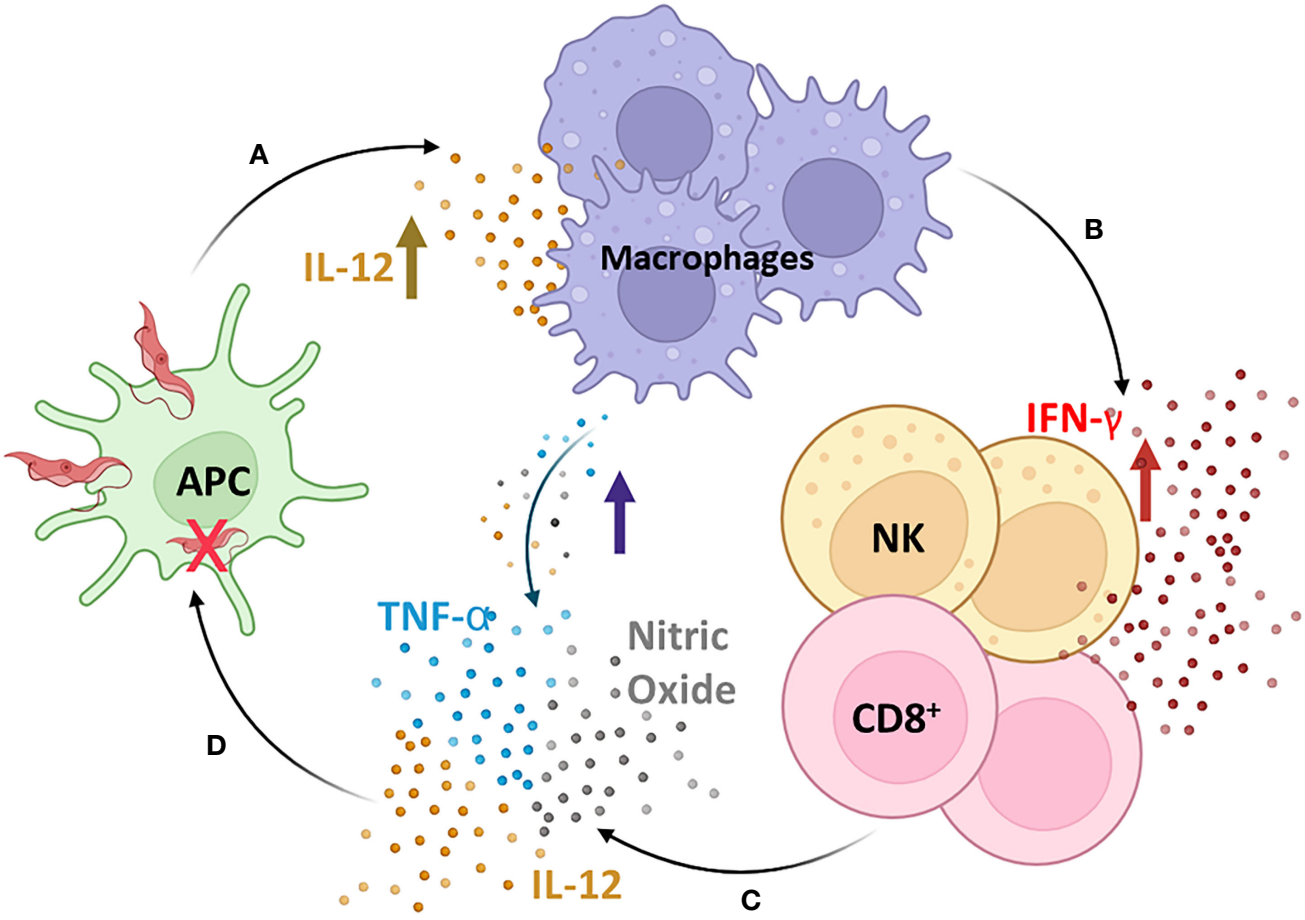
The cascade of induction of the innate immune response. *T. cruzi* initiates a cascade of events that trigger the synthesis of IL-12 by macrophages **(A)**, which is a crucial mediator of IFN-γ production through Th1 and Natural Killer (NK) activation **(B)**. IFN-γ plays a crucial role during *T. cruzi* infection by increasing the production of IL-12, TNF-α, and nitric oxide (NO) by macrophages **(C)**, which are cytotoxic to intracellular microorganisms and exert trypanocidal activity, inhibiting the development of *T. cruzi* **(D)**. Moreover, IFN-γ stimulates T-cytotoxic lymphocyte activation, a central mechanism for systemic and antigen-specific protection against *T. cruzi* intracellular infection. APC, antigen-presenting cells; NK, natural killer cells; CD8^+^, CD8+ T lymphocytes. Figure created by the author on the biorender.com platform, inspired by an Oswaldo Cruz Foundation website publication.

Although the adaptive immune response has been considered for many years to be the most relevant in building the balance between protective and pathogenic events during chronic infection of Chagas disease, several studies have demonstrated the importance of the innate immune response as a significant factor in this issue, mainly in the process (154). During the acute phase, cells of the innate immune system play a pivotal role in controlling the infection by suppressing parasite replication. The classic mechanism proposed is that *T. cruzi* initiates a cascade of events that trigger the synthesis of IL-12 by macrophages, which is a crucial mediator of IFN-γ production through Th1 and NK activation (158, 159) (**Figure 2**). IFN-γ plays a crucial role during *T. cruzi* infection by increasing the production of IL-12, TNF-α, and nitric oxide (NO) by macrophages. The NO is cytotoxic to intracellular microorganisms and exerts trypanocidal activity, inhibiting the development of *T. cruzi* (160–162) (**Figure 2**). Moreover, IFN-γ stimulates T-cytotoxic lymphocyte activation, a central mechanism for systemic and antigen-specific protection against *T. cruzi* intracellular infection (163, 164) (**Figure 2**).

**Figure 3:**
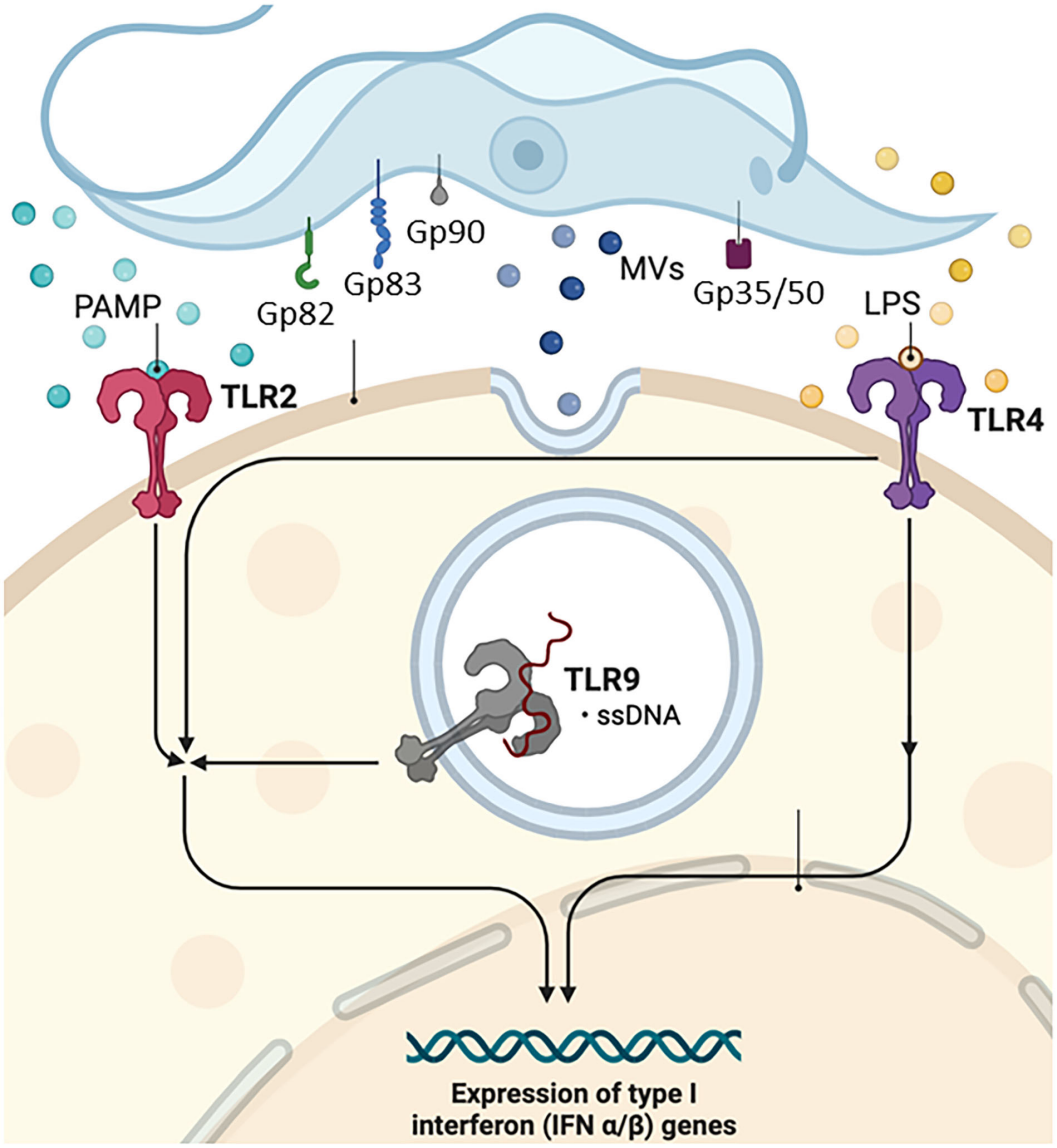
Complement system. Gp, glycoprotein; Tc, *T. cruzi* molecule; TLR, Toll-type receptors; MAC, complement membrane attack complex; MBL, mannose-binding lectin; MVs, microvesicles; T-DAF, Trypanosoma-decay accelerating factor. Figure created by the author on the biorender.com platform, inspired by an Oswaldo Cruz Foundation website publication.

One of the first lines of defense is the complement system, which consists of more than 35 plasma and cell surface receptors/regulators. After interaction with the pathogen, it can be activated by three pathways of the protease cascade: classical (CP), alternative (AP), and lectin (LP), resulting in inflammation, opsonization (phagocytosis), and lysis of pathogens (**Figure 3**) (165–167). Although these pathways differ in the initial steps of their respective cascades, all three converge to produce a C3 convertase and then a C5 convertase, forming a membrane attack complex (MAC) and subsequent pathogen lysis (167). The main mechanisms involved in the ability and capacity of trypomastigotes to avoid complement action are as follows: *T. cruzi* calreticulin (TcCRT), which is a 45-kDa calcium-binding protein, primarily located in the endoplasmic reticulum (ER) and expressed in infective trypomastigotes. After infection, TcCRT translocates to the emerging area of the flagellum on the plasma membrane surface (168–170). This protein can bind to host mannose-binding lectin (MBL) and interacts with ficolins, preventing C4 conversion to C4b and C1q and interfering in the activation of the CP and LP (170–172). TcCRT has also been shown to internalize parasites into mammalian cells (171). Another mechanism is *T. cruzi* Complement Regulatory Protein (TcCRP): the protein TcCRP, also called Gp160, is a 160-kDa glycoprotein anchored into trypomastigote membranes that can bind to C3b and C4b, preventing the assembly of proteolytically active C3 convertase, thus inhibiting the formation of CP and AP complement C3 convertase (168, 170, 173). The *T. cruzi* complement C2 receptor inhibition trispanning (TcCRIT) is a 32-kDa transmembrane protein mainly expressed in trypomastigotes. TcCRIT has amino acid sequence homology with the C4 beta-chain, the binding site of C2. Thus, it blocks C2 cleavage by C1s or MASP-2 into C2a and prevents C3 convertase formation, thus regulating the activation of the LP and CP (174–177) mediating cell lysis, allowing survival and cell invasion by the parasite. The glycoprotein 58/68 (Gp58/68) is a glycoprotein of 58 kDa (non-reduced) and 68 kDa (reduced) (178), expressed on the parasite surface or released by trypomastigotes. It is a part of the fibronectin/collagen receptor of *T. cruzi* and plays an important role in the interaction of *T. cruzi* with mammalian cells (178, 179). This protein was able to inhibit the formation of cell-bound and fluid-phase C3 convertases (178) to allow the parasite to evade AP complement activation by preventing the initial association of factor B (FB) with surface-fixed C3b (167, 178). The trypomastigote decay-accelerating factor (T-DAF) is an 87- to 93-kDa glycoprotein expressed on the surface of metacyclic trypomastigotes of *T. cruzi*. T-DAF mimics the activity of the complement regulatory protein DAF and regulates the activation of the AP, CP, and probably the LP of the complement by interfering with the assembly efficiency of C3 convertases (180, 181), which is essential for the escape of *T. cruzi* from complement activation and lytic effects (167).

Extracellular vesicles, whether microvesicles (MVs) or exosomes, shed by pathogens, transfer virulence factors and biomolecules to host cells, thereby altering the host’s susceptibility to infection (177) (**Figure 1**). Cestari et al. (2012) (176) and Wyllie and Ramirez (2017) (177) described a mechanism of complement immune evasion by *T. cruzi* through the induction of membrane-derived vesicles from host cells by metacyclic trypomastigote forms and the consequent secretion of these MVs. MVs are 100- to 1,000-nm molecules produced by outward budding and fission of the plasma membrane, released by cell blood, immune system, epithelial, and endothelial tissues, among others. They are an integral part of the intracellular microenvironment and act as regulators of cell-to-cell communication (182, 183). The complement system is a fundamental immune response component, recognizing and eliminating pathogens. *T. cruzi* may evade immediate immune responses by inhibiting the complement system, promoting its survival in the host environment (177). The infective form of the parasite induces the release of MVs from immune cells in a calcium-dependent manner at the beginning of infection. These MVs bind LP (177) and CP C3-convertase complexes on the surface of *T. cruzi* inhibiting complement-mediated lysis and favoring invasion and infection of host cells (176). Interestingly, MVs derived from different parasite strains (TcI and TcII) did not alter complement resistance and the invasion process (177). These studies provide valuable insights into the complex interplay between the parasite and host cell-derived MVs, establish a communication network that allows it to manipulate host cell functions, modulate immune responses, and potentially escape immune surveillance, contributing to the chronicity of *T. cruzi* infection and the establishment of persistent parasitemia (176).

According to Quijano-Hernandez and Dumonteil (2011) (184), the protective immune response against *T. cruzi* requires the activation of a Th1 immune profile with stimulation of T-cytotoxic cells and B-lymphocytes to secrete parasite-specific immunoglobulins (185). Furthermore, the control of parasite infection spread has also been associated with NK cells (186). Sathler-Avelar et al. (2006) (187) suggested that NK cells could protect against early *T. cruzi* infection, comparing the cytokine profile of patients with Chagas disease with healthy uninfected children. Vitelli-Avelar et al. (2005) (188) analyzed the frequency of NK cells in the peripheral blood of patients with Chagas disease, both of which were particularly high in indeterminate patients. These authors suggested that increased circulating NK cells may protect the host from morbidity during the chronic phase.

It has been proposed that *T. cruzi* DTU promotes diverse host immune responses and affects disease progression (**Table 1**). Similarly, anti-inflammatory and pro-inflammatory cytokines may play a pivotal role during infection and the differential regulation of cytokine synthesis in developing the Chagas cardiomyopathy or indeterminate clinical status (31, 188–190). It has been described that the lower expression of IL-10 is associated with the development of chagasic cardiomyopathy (190), and a higher expression was observed in patients with the indeterminate form (188–191), suggesting that IL-10 contributes significantly to parasite control without severe damage, regulating immune response during chronic Chagas disease. Magalhães et al. (2015) (119) showed that while TcI DTU induced IL-10 production and higher monocyte activation, TcII DTU led to lower monocyte activation but a higher inflammatory profile. TGF-β was demonstrated to play a role in regulation during the acute phase of Chagas disease, whereas it was shown to be a potent inhibitor of the effects of macrophage-activating cytokines, such as IFN-γ (192). IFN-γ and TNF-α, inflammatory cytokines, and cytotoxic cells have been correlated with tissue damage and the severity of chronic Chagas disease (190, 193–195) in cardiac patients, suggesting a role for pro-inflammatory monocytes in developing this disease. Guedes et al. (2012) (35) showed that higher levels of IL-17 were associated with less aggressive chagasic cardiomyopathy.

Distinct *T. cruzi* DTUs may exhibit variations in parasitemia, immune responses, and tissue pathology in their hosts (89–91, 93). Moreover, these parameters may differ in co-infections compared to *T. cruzi* mono-infections (89). In a study comparing the biological behavior of TcII and TcVI DTUs conducted by Rodrigues et al. (2010) (89), it was demonstrated that TcII presented reduced parasitemia in mice compared to TcVI. TcVI induced higher parasitemia, increased the systemic release of pro-inflammatory mediators, and induced higher mortality during the acute phase of the disease. Co-infection with TcII and TcVI results in intermediate parasitemia, heart parasitism, and mortality. This finding correlated with a decrease in inflammatory cells, suggesting that the *T. cruzi* co-infected animals exhibited a less intense inflammatory reaction in the heart. These data indicate that *T. cruzi* co-infection can trigger both protective inflammatory immunity and regulatory immune mechanisms that attenuate the damage caused by inflammation, consequently reducing disease severity (89). However, when the biological behaviors of TcII and TcIV were compared in infected mice, TcIV showed lower virulence and fewer inflammatory processes than TcII. This includes significantly lower parasitemia levels and a lower frequency of organs with inflammatory processes (90). Magalhães et al. (2019) (93) observed that TcIV induced a regulated profile in human monocytes, whereas TcV induced an inflammatory profile. Differences in the host’s cellular immune response were noted considering the different DTUs and phases of *T. cruzi* infection (91). In an experimental model using dogs, Duz et al. (2014) (91) proposed that, in the acute phase, TcI could go unnoticed by peripheral blood mononuclear cells, enabling faster parasitization of target organs. In the chronic phase, TcI presents characteristics of inflammation, with higher levels of IL-4 observed in the peripheral blood. TcII DTU triggered the production of IFN-γ and IL-4 in the acute phase, and significant heart inflammation and fibrosis were observed in the chronic phase. Differences in humoral immune response were also associated with different DTUs during acute and chronic *T. cruzi* infection in mice. TcI is more efficient in overexpressing specific antiparasitic IgG subclasses (IgG, IgG1, IgG2a, and IgG2b) than TcII and TcV (88). This finding was recently corroborated by Silveira-Lemos et al. (2021) (81), who demonstrated that distinct *T. cruzi* genotypes influence the phenotypic and functional features of the host immune response.

The host genetic background plays an important role in the course of infection, such as the genetic variability among the six different DTUs of *T. cruzi* that interact differently with the host and their eco-epidemiological effects. The immune response is decisive throughout the entire process. The cytokine profiles switch between anti-inflammatory and pro-inflammatory cytokines, which may be relevant in determining chronic patients’ clinical presentation and disease outcomes. These results show that the progression of human Chagas disease from asymptomatic to severe forms is related to a lack of adequate immune modulation.

## 5 Pathogenesis and DTU

The clinical course of Chagas disease is polymorphic, ranging from asymptomatic to severe chronic cardiovascular or gastrointestinal involvement. After parasite transmission, trypomastigote forms of *T. cruzi* can invade different cell types, including macrophages, muscle cells, and fibroblasts. Parasitic amplification into the cytosol results in intense tissue parasitism and blood parasitemia during the acute phase. The symptoms in this phase are generally mild, except in immunosuppressed patients and children who may develop cardiomyopathy and encephalomyelitis (196, 197). After the acute phase, parasitemia decreases in the chronic phase of the disease, starting with the asymptomatic or indeterminate form, where no clinical symptoms are observed. Most individuals remain in this phase of infection throughout the rest of their lives. Approximately 30% of these patients develop chronic phase disease with cardiac and/or gastrointestinal symptoms. This involvement may be severe and a determinant of the morbidity of the disease (32, 198, 199).

Cardiomyopathy is one of the most important clinical changes in patients with Chagas disease in the chronic phase due to the high frequency with which it develops (in 20%–30% of infected individuals), in addition to its severity, morbidity, and mortality (200). The classic pathology of chronic Chagas cardiomyopathy includes myocarditis accompanied by myocytolysis, myofiber hypertrophy, and interstitial fibrosis (201). In general, only focal areas of inflammation are found in the hearts of patients with chronic Chagas disease. Inflammatory infiltrates mainly comprise T cells, macrophages, eosinophils, plasma cells, and mast cells (200). Fibrosis in chronic Chagas disease exhibits a unique distribution pattern, often surrounding and involving individual myocardial fibers. This feature distinguishes it from the fibrosis patterns in idiopathic dilated cardiomyopathy (202, 203). Moreover, fibrosis in Chagas disease can also be characterized by intrafascicular deposition of collagen (204, 205), which causes disorganization and isolation of cardiomyocytes and contributes to the electrocardiographic changes observed in a canine model and in humans (206). The host innate and adaptive immune responses may contribute to cardiac damage and increase the risk of heart failure through the induction of inflammation, fibrosis, and oxidative stress injury, leading to myofibril rupture, cardiomyocyte necrosis, microvascular dysfunction, autonomic dysfunction, cardiac hypertrophy, and fibrosis (200). Duz et al. (2014) (91) evaluated the immune response and cardiac lesions in dogs infected with Colombian (TcI) and Y (TcII) strains of *T. cruzi*. The authors demonstrated that in the chronic phase, the inflammation triggered by the Y strain was balanced by tissue rearrangement and fibrosis, whereas infection with the Colombian strain showed more significant inflammation and a lower degree of cardiac fibrosis. Ferrer et al. (2014) (207) analyzed the impact of different *T. cruzi* isolates obtained from Argentine patients (TcI, TcV, or TcVI) on cardiac tissue from *T. cruzi*-infected CF1 mice and showed that the degree of myocarditis ranged from minimal to mild for TcV and TcVI to moderate to intense for TcI. *T. cruzi* DTUs define the pattern of inflammatory mediators in heart tissue and may contribute to the magnitude of cardiac pathogenesis (208).

Controversies surround the question of the relative contribution of autoimmunity and the presence of the parasite to the development of chronic *T. cruzi* infection. Persistent inflammation and divergence between low parasite load in the tissue and the severity of the lesions observed during the chronic phase support this autoimmune etiology (209, 210). The autoimmunity hypothesis suggests that tissue damage leads to an immune reaction against self-proteins. This theory has been considered a key mechanism to explain the tissue damage observed in the chronic phase of the Chagas disease, even in the absence of parasites in the affected tissues.

However, despite the wide acceptance of autoimmunity in the etiology of Chagas disease, several studies have suggested a strong association between parasite load and disease severity (211–214). Even if anti-self-immune responses are induced in these models of Chagas disease, these responses are insufficient to generate disease without local parasitic infection. The direct pathogenic role of the parasite has gained strong support from very sensitive new immunohistochemical techniques and applications of PCR, which show a strict correlation between the presence of the parasite and tissue lesions (210, 215, 216).

The circulation of distinct genotypes of *T. cruzi* within infected triatomines suggests complex interactions among various parasites, leading to diverse infection patterns (125, 217). This genetic diversity may account for regional variations in the incidence of the cardiac and/or digestive forms of the disease. However, the distinct clinical manifestations of Chagas disease can arise from factors beyond the genetic diversity of *T. cruzi*, such as the interaction between the parasite and the host (60, 125, 190, 218).

The varied symptoms of Chagas disease likely result from differences in the immune response among individuals, which effectively control parasitic levels and limit organ damage, while inefficient responses fail to control parasite proliferation, resulting in persistent inflammation and severe clinical manifestations (219). Parasites selectively form multiclonal populations in different individuals, giving rise to stable lineages. According to the “clonal histiotrophic model” of Chagas disease, the polymorphism of biological factors allows distinct *T. cruzi* clones within a lineage to exhibit tropism for various tissues, which also contributes significantly to the clinical outcome of the disease (220, 221). Classic studies have demonstrated that the Y strain (TcII) is associated with high parasitism of the spleen, liver, and bone marrow in addition to increased virulence in the acute phase of the infection, whereas the Colombian strain (TcI) presents cardiomyotropism and pathogenicity in the chronic phase (78, 222, 223). The *T. cruzi* clone Sylvio-X10/4, belonging to the TcI DTU, also produces myocarditis in C3H/He mice, similar to that observed in human chronic Chagas disease caused by the Colombian strain (TcI) (224). On the other hand, different groups have pointed out that the Y strain (TcII) produces inflammation at the cardiac level in mouse models (225), and that infection with the CL (TcVI) strain results in high parasitism of countless cell types, indicating that the *T. cruzi* strain is pan-infective and does not affect only the heart tissue (226). In a canine model, the Y strain (TcII) triggers a more robust immune response during the acute phase of infection than the Colombian strain (TcI) (127). Colombian infection (TcI) shows characteristics of inflammation during the chronic phase. It is possible that this strain can escape the host’s acute immune response and parasitize the organs faster, while the Y strain (TcII) triggers a specific immune response during the acute phase, helping to control myocardial lesions in the early chronic phase (91). A more recent study realized by Reis Machado et al. (2014) (92) shows that the animals infected or reinfected with the Colombian strain (TcI) had a significantly higher expression of IFN-γ, both in the cardiac tissue and in spleen cells when compared to groups infected with the Y strain (TcII).

De Diego et al. (1998) (227) found qualitative and quantitative histopathological differences in studying *T. cruzi* genotypes belonging to different DTUs. Genotype 39 (TcV) was detected in infected mice’s skeletal muscle, spleen, liver, and heart. In genotype 20 (TcI), heart inflammatory foci were small. In genotype 19 (TcI), skeletal and cardiac muscle fibers were the most parasitized, demonstrating that differences can occur between *T. cruzi* strains in the same subgroup (206). In rats, infection with JG (TcII) and CL Brener (TcVI) revealed differential tissue tropism among the strains. While the JG strain (TcII) occurs in the cardiac muscle, CL-Brener (TcVI) is detectable in skeletal muscles and other organs (129). In a study of Colombian patients, TcII was detected in the heart, causing chronic chagasic cardiomyopathy (CCC). In contrast, TcI was found in the muscular layer of the esophageal tissue, along with lymphocytic infiltrates and interstitial fibrosis (126). In D’Avila et al. (2009) (228), the authors did not find a correlation between the genetic profiles of *T. cruzi* isolates and different clinical forms of Chagas disease. Likewise, Del Puerto et al. (2010) (64) analyzed blood samples from 306 Bolivian patients with Chagas disease in the chronic phase and found no associations between *T. cruzi* DTUs and the clinical manifestation of the Chagas disease. The differences between the findings indicate that histopathological studies can produce different results according to genetic variations between *T. cruzi* strains and the experimental model or type of clinical study performed.

It is well known that *T. cruzi* can change virulence and tissular tropism over time to maintain its infectious properties, depending on factors from the host (229, 230). Many groups have not successfully correlated the parasite’s genetic variability with the disease’s clinical characteristics since most studies on *T. cruzi* isolates and the parasite’s growth in laboratory animals or *in vitro* cultures have a high chance of clonal selection. Often, parasite populations in the heart, skeletal muscle, liver, or spleen differ from those found in the host’s blood (231). The presence of different *T. cruzi* DTUs with different tropisms in the same host originated from distinct populations of parasites in diverse tissues, even when infected with a particular strain. Some studies have demonstrated different *T. cruzi* genetic groups in the bloodstream and heart tissue of the same host (221, 232–234). These findings indicate the need to carry out studies with tissue and bloodstream samples from patients with Chagas disease, to compare all *T. cruzi* genotypes present in the same individual, as they will probably not be the same.

Differential tissue distribution of *T. cruzi* populations has been detected in male BALB/c mice infected with two clonal stocks (124). In short, using a quantitative assay to estimate the proportion of each clone in the profiles, 80% predominance of the strain Col.1.7G2 (TcI) concerning the strain JG (TcII) was observed in the rectum, diaphragm, esophagus, and blood of the infected mice, in contrast with up to 90% preponderance of the strain JG (TcII) in the heart of the same mice. Curiously, the strain Col.1.7G2 (TcI) used in this study was obtained from a patient with cardiac Chagas disease and showed tropism for smooth muscles in mice, while strain JG (TcII), obtained from a patient with the digestive form of the disease showed cardiotropism. Hence, the behavior of parasites within disparate reservoirs may exhibit variations (124, 232). Vago et al. (2000) (125) showed that parasites possessing distinct genetic profiles can be discerned in separate tissues, such as the esophagus and heart, within the same patient.

Studies that used an initial inoculum containing strains of different *T. cruzi* DTUs have detected the predominance of one strain over the others (120, 124, 127, 235, 236). The survival of only one isolate in a given tissue could be related to its ability to escape from the immune system or to a selective process within host cells (127). Thus, the distribution of different parasites in host organs could be influenced by the interactions between parasites of different groups, and the presence of a specific population of *T. cruzi* in tissues could be related to distinct clinical manifestations of the disease. The diversity of parasites triggers a more complex immune response that restricts the more susceptible parasites to specific organs or even removes them from the host. A particular population of *T. cruzi* could infect different tissues within the host, but their presence within a particular organ would be determined by other parasites in the co-infection and by interactions with the host’s immune system (236). Given the above, the parasite strain association is more important than the DTU, and the composition of the infecting parasite population plays an important role in the host response against *T. cruzi* as well as in the outcomes and severity of infection (89).

As final remarks, despite these findings, it is still difficult to establish a correlation between the genetic diversity of *T. cruzi* and the clinical manifestations of Chagas disease. Although there was some experimental support for the differential tissue tropism of *T. cruzi* strains (126), other reports failed to link parasite genetics to the clinical forms of Chagas disease (41, 220, 237). The genetic aspects of parasites may play an essential role in determining which host tissues will be infected and thus influence the pathogenesis of the disease. However, it is important to carry out studies that evaluate the behavior of different isolates in co-infections (115), considering that this interaction could modify the intrinsic behavior of individual isolates, changing the origin of the infection in Chagas disease. Developing tools capable of accessing intra-group genetic and gene expression variability is also necessary.

## 6 Immunosuppression and reactivation of the Chagas disease

Reactivation in Chagas disease occurs when *T. cruzi* escapes from the host’s immune system, resulting in multiplication, tissue inflammation, and increased blood parasitism in chronic infections (55, 238). This process is probably due to an imbalance in the relationship between the parasite and host when the cellular immune system is affected, where parasite persistence has been associated with myocarditis progression (61). In most cases, immunosuppression modifies pre-existing diseases, leading to developing or reactivating opportunistic infections, which present special and often severe clinical features (239).

Cases of reactivation of infectious parasitic diseases have been commonly recorded, such as Chagas disease, visceral leishmaniasis, and toxoplasmosis, in transplant patients treated with immunosuppressives in organ transplants or blood transfusions (240, 241) and in cases of co-infection with human immunodeficiency virus (HIV) (54, 242).

Since the 1990s, there have been reports of reactivation of chronic Chagas disease in individuals infected with HIV (238, 243–245). In these patients, *T. cruzi* most commonly affects the central nervous system (75%–80% of cases) and is often associated with cardiomyopathy (246, 247). The clinical manifestations included acute meningoencephalitis, fever, focal neurological deficits, and histopathological examination revealing inflammation with amastigotes in glial cells and neurons (248). Individuals may also present with cardiac symptoms (25% to 44% of cases), such as heart failure and arrhythmias.

Despite the fact that Chagas disease is a lifelong infection, the anti-trypanosomal therapy for infected people during the chronic phase of the disease is not clearly effective and remains a challenge (249). Heart transplantation is a therapeutic option for those patients with advanced heart failure refractory to medical therapy. Reactivation of Chagas disease is a common finding under immunosuppressive conditions, such as AIDS, autoimmune diseases, cancer (and the chemotherapy used to treat it), and, obviously, pharmacological immunosuppression to avoid allograph rejection (250–252).

Severity of the acute phase of *T. cruzi* infection is closely related to aggressive immunosuppression. Type I T helper immune response is an important mechanism involved in the *T. cruzi* infection (185). It is well established that a high immunossupression is able to modify the cytokine profile of type I T helper lymphocytes to Th2-type response. This environment constitutes a favorable condition for exacerbation of the disease and its symptoms including fever, anorexia, myalgia, diarrhea, panniculitis, myocarditis, meningoencephalitis, and encephalitis (252). In this context, some *T. cruzi* DTUs seem to contribute to increase the risk of certain morbidities in comparison to others. On the other hand, since host–pathogen interactions are complex, with several unknown aspects influencing the fate of infections, the interplay between the pathogen and the host’s immune response must be critical in determining disease evolution (252). The alignment between the distribution of *T. cruzi* DTUs and disease severity has led to a general association of TcI with oral transmission and severe acute cases in the North and Central Americas and the Amazon region in Brazil; TcIII and TcIV associated with oral transmission and acute cases in the Amazon region in Brazil and Venezuela, respectively (252).

Considering the natural history of Chagas disease, approximately 30% of *T*. *cruzi*-infected individuals develop progressive destruction of the myocardium, which causes chronic chagasic cardiomyopathy. The progress of the inflammatory process will debilitate the patient and enhance the chances of requiring a heart transplant, which becomes the only treatment option. However, reactivation of the disease may occur due to concomitant immunosuppressive therapy (249, 253, 254).

It has been reported to occur in 26.5% to 42.9% of patients who underwent cardiac transplantation (255, 256), ranging from asymptomatic to the presence of symptoms such as fever, subcutaneous involvement, and myocarditis (257). Sánchez-Valdéz et al. (2018) (258) documented the development of non-proliferating intracellular amastigotes of *T. cruzi* (dormancy state), which were uniquely resistant to extended drug treatment and could re-establish infection after as many as 30 days of drug exposure. This finding may help understand the failure of highly cytotoxic compounds to completely resolve the infection in chronic patients and make the relationship between immunosuppression and Chagas disease worrying.

Several teams have investigated the correlation between *T. cruzi* DTU and Chagas disease reactivation (28, 54, 259–262). Burgos et al. (2008) (263) identified mixed infection by *T. cruzi* I and *T. cruzi* IV/V in blood, but only *T. cruzi* I was found at the site of CNS reactivation. Costales et al. (2015) (264) and Inga and Oliveira (2019) (265) related the reactivation of the disease to the parasites of the TcI, as previously found in cases of reactivation by HIV co-infection (263). Cura et al. (2012) (266) demonstrated that the distribution of parasites in tissues and blood differs between immunocompetent patients and cardiac transplant patients with reactivation. This result may reflect the unmasking of mixed infections below the detection level before reactivation. The authors showed that TcI was more frequent in the bloodstream and cardiac tissues from immunosuppressed patients, while TcI and TcII/VI were detected in skin biopsy slices in a kidney transplant patient. Recently, Marcon et al. (2022) (262) investigated the DTU present in the blood of *T. cruzi*/HIV co-infected patients with antiretroviral infection in Brazil and showed that the majority of them were infected with TcII and one case had mixed infection (TcII and TcV/TcVI).

Experimental models have shown that the severity of pathogenesis and tissue parasitism during chronic reactivated disease resembles the symptoms presented in the acute phase of infection. Immunodeficient mice infected with *T. cruzi* showed higher parasitemia and myocardial parasitism with inflammation than mice infected with *T. cruzi* without immunosuppression (267). Santos et al. (2010) (86) observed a distinct pattern, demonstrating that the *T. cruzi* genetic lineage, in combination with specific treatment with benznidazole, plays an important role in the reactivation of infection in immunosuppressed mice. The authors observed that animals infected with TcII showed reactivation in only 5% of cases. In comparison, those infected with TcI clones showed reactivation in greater than 51% of the animals when immunosuppressed with cyclophosphamide. These data reinforce the strong presence of TcI DTU in cases of Chagas disease in clinical studies.

Nonetheless, in reactivated Chagas disease, several *T. cruzi* DTUs have been detected by molecular methods, as previously discussed. It is currently impossible to establish a strict correlation between specific DTU and Chagas disease immunosuppression (28, 54, 61, 259–261). The DTU identified seems to reflect the more common genotype in a defined geographic region. Sales-Campos et al. (2014) (127) observed that the behavior of strains with the same DTU can be different in immunosuppressed mice. These data reinforce the idea that the composition of the infecting parasite population plays an essential role in host–parasite interactions (89, 96, 268).

The biological and genetic heterogeneity of circulating strains of *T. cruzi* can make it challenging to interpret the different manifestations of the disease and determine the correlation between DTU and specific clinical traits. Burgos et al. (2010) (61) showed distinct *T. cruzi* signatures in cardiac explant specimens and blood samples obtained from a cohort of 16 Argentinean patients with cardiac disease who underwent transplantation. TcI DTU was observed in three explant samples, and TcII, TcV, and TcVI were observed in five samples. Post-heart transplantation follow-up identified TcI in five patients and TcII, TcV, and TcVI in seven patients. TcI, TcV, and TcVI were detected in skin chorioma specimens, and TcV and TcVI were detected in samples obtained from patients with myocarditis reactivation.

However, it should be considered that this is a complex process involving several host and parasite factors. It is reasonable to suppose that the immunological condition of the patient, whether associated or not with the reactivation of the infection, and the strain of the parasite may have an important role during the course of the disease.

Considering the data presented, it is reasonable to suppose that the immunological condition of the patient, whether associated or not with the infection’s reactivation and the parasite’s strain, may have an important role during the Chagas disease reactivation process (261). Thus, considering the large number of infected patients, the increasing cases of immunosuppression, and the severity of the disease in this situation, we emphasize that the role of genetic and biological aspects of *T. cruzi* on Chagas disease reactivation needs to be further explored in basic and clinical research.

## 7 Influence of *T. cruzi* DTUs in congenital transmission of the Chagas disease

Congenital transmission has emerged as a significant pathway for the dissemination of *T. cruzi*, presenting a global challenge that endures even in nations equipped with robust vector control programs and established blood banks (269, 270), and represents the predominant mode of transmission in non-endemic regions (271–274).

A comprehensive meta-analysis by Howard et al. (2014) (275) assessed the frequency of congenital transmission, revealing a 5% infection rate among chagasic mothers. The risk of vertical transmission exhibits considerable disparity, ranging from 0.75% to 28.6% across diverse geographic regions (275). The robustness and efficacy of congenital infection screening services significantly influence this aspect (275). Additionally, it has been proposed that genetic *T. cruzi* diversity is a pivotal factor in shaping this process (276). In support of this hypothesis, Cencig et al. (2013) (277) explored the impact of infection with three *T. cruzi* DTUs (TcI, TcII, and TcVI) on fertility, pregnancy, and congenital transmission. The authors observed that the parasite induced significant damage in the acute phase, irrespective of genotype, whereas in the chronic phase, the primary symptom was delayed uterine growth. Vertical transmission was detected in the acute phase of the infection, rare in the chronic phase, and associated with TcII and TcVI. Nevertheless, Alkmim-Oliveira et al. (2013) (278) experimentally assessed the potential for congenital transmission of TcI and TcV in female BALB/c mice. They observed that both genotypes could be transmitted to the fetus at rates of 58.1% and 46.6%, respectively. Females infected with TcI exhibited a lower average number of pups per female (4.8) than those infected with TcV (8.3).

A distinct association between transplacental transmission and specific *T. cruzi* DTU is not evident in human infections. These data substantiate the hypothesis that the results reflect the most prevalent DTU within these regions, given that most DTUs have been discerned in human cases of congenital infection. For example, in Bolivia and Argentina, TcV predominantly constituted the detected DTU, although TcII and TcVI have also been documented (278–282). These genotypes are recurrently linked to vertical transmission across the Southern Cone of America (278, 283–286). TcI has been documented in Mexico, Central American countries, and Colombia (287–289) and has been detected in congenital infection cases in Argentina and Chile (285, 286). Herrera et al. (2019) (289) revealed a higher frequency of TcII-TcV-TcVI than TcI in Honduras and Mexico, implying that the prevalence of these genotypes in these regions may be more extensive than previously acknowledged.

Moreover, several studies have identified that mixed *T. cruzi* infections involving different DTUs or haplotypes within the same DTU are frequently detected (281, 282, 289, 290). A significant aspect of mixed infections is the detection of genetic profiles in infants that differ from those observed in the mother (282, 290). This suggests that the occurrence of polyclonal strains is common, highlighting that the dynamics of the *T. cruzi* population are profoundly influenced by both the host and the environment.

In Brazil, genotyping of *T. cruzi* from congenital infections is limited, likely reflecting the country’s deficient structure of congenital infection surveillance and patient management (291). Bittencourt et al. (1985) (292) characterized isoenzymes of parasites isolated from mothers and infants from Bahia state. Their observations revealed that, except for one triple heterozygous GPI pattern, all the identified patterns were associated with zymodeme 2. This suggests the probable occurrence of TcII, representing the primary genotype infecting patients in the central and southern regions or, secondarily, TcVI. Data from our research team on Chagas disease in the Jequitinhonha Valley, Brazil, MG, coordinated by Marta de Lana, PhD, support this information through the genotyping of strains obtained from mothers and children in the northwest region of Minas Gerais. The results revealed identical molecular profiles between the paired strains, all isolates were identified as TcII, and similar ones were found most frequently in this region (67). Llewellyn et al. (2015) (290) also detected TcII in cases of congenital infection in the Goias state. TcIV was first identified in association with a potential case of congenital infection in the northern region of Brazil, specifically in the state of Pará (293). This was linked to a maternal infection during the oral transmission outbreak. The authors also documented a case associated with TcI, and the findings once again reflected the DTU most frequently observed in transmission cycles within the region (39). TcIII and TcIV were less often associated with congenital infections, likely due to their lower occurrence in *T. cruzi* domestic transmission cycles.

The studies mentioned above showed that *T. cruzi* genetic diversity alone is inadequate to elucidate the incidence of congenital infection, even more so considering the intense molecular and biological polymorphisms observed in the species and that other factors play a crucial role in the success of this infection. The probability of vertical transmission by *T. cruzi* is affected by the infection stage, reinfection, maternal immune response, placental factors, and parasite characteristics such as infectivity, virulence, and tissue tropism (294–298).

Within the framework of *T. cruzi* subpopulations, certain studies have revealed that different strains exhibit distinct capabilities to infect placental tissues. For instance, Andrade (1982) (299) emphasized the significant influence of parasite strain on congenital *T. cruzi* infection. The presence of parasites in the placenta was infrequent and limited in animals infected with Type I and II strains (Y, Peruvian, and Honorina). In contrast, it was intense and frequent in those infected with the Colombian strain (Type III). The authors also noted that the probability of placental infection appears more plausible during peak parasitemia in the acute phase than in the chronic phase. Medina et al. (2018) (300) demonstrated notable variations in the infectivity and pathogenicity of two *T. cruzi* strains within the placental cells of mice. This underscores the significance of the intrinsic characteristics of *T. cruzi* stocks and their interactions with the host in determining the success of congenital infections. These findings indicated that the VD strain (TcVI) exhibited higher infectivity and pathogenicity than the Y strain (TcII).

However, few studies have assessed the behavior of DTUs in experimental congenital infections that have been conducted, often confined to one or two *T. cruzi* genotypes. To comprehensively understand the influence of the intrinsic characteristics of the parasite on congenital transmission, it is necessary to investigate a more extensive array of strains from all DTUs, encompassing their diverse biological properties. Furthermore, it should be noted that some studies have shown important genetic variability within the same DTU associated with congenital transmission (283, 289, 290). Burgos et al. (2009) (283) showed different minicircle signatures in *T. cruzi* stocks belonging to the same lineages in Chaco Province, Argentina. Herrera et al. (2019) (289) confirmed that analyzing intra-DTU genetic variability may provide more informative insights into its correlation with congenital infection. In their study on *T. cruzi* stocks isolated from the umbilical cord blood of newborns, the authors observed the presence of TcI, TcII, TcV, and mixed infections circulating in Argentina, Honduras, and Mexico. Through detailed haplotype sequencing of isolated parasites, they observed the circulation of identical haplotypes in these countries at varying rates. For the first time, a strong association was observed between parasite haplotypes and congenital infection, suggesting that identifying parasite haplotypes in pregnant women could predict the vertical transmission of *T. cruzi* in this geographic area.

Remarkably, the interaction between *T. cruzi* subpopulations and the host often elicits tissue and cellular responses that can significantly influence the course of congenital infection (295). Several studies have reported the modulation of gene expression in the placenta due to infection. Castilho et al. (2017, 2018) (301, 302) observed alterations in the expression of genes associated with innate immune response during *ex vivo* infection of human placental explants. Initially, they investigated the response of human placental chorionic villi explants to infection with *T. cruzi* (Y strain) and *Trypanosoma gondii.* They found that *T. cruzi* infection is linked to the expression and activation of TLR-2, while *T. gondii* infection is mediated by TLR-4 and TLR-9. Inhibition of these receptors increases the DNA parasite load in placental explants.

Additionally, *T. cruzi* induces increases in the expression and secretion of the cytokines TNF-α, IL-1β, IL-6, IL-8, and IL-10, whereas *T. gondii* increases the expression of TNF-α and CXCL8 but only elevates the secretion of IL-8 (301). Additional studies have revealed that genes involved in complement regulation and function, such as CD46 and C1q, were upregulated in *T. cruzi* infection. Furthermore, TLR7 and TLR8 showed increased expression, along with the cytokine IL-6, dependent on the parasite burden (302). The immune response profile elicited by the parasite likely affects its capacity to establish and regulate damage (303). Therefore, the local response of the placenta to infection is intricately linked to the risk of congenital transmission and can be influenced by factors such as the host’s strain, parasite load, and genetic background (296–298, 304). Juiz et al. (2017) (305) also demonstrated that *T. cruzi* infection regulates genes associated with innate immunity and the response to interferon-gamma when studying VD and K38 strains in C57Bl/6J mice. The authors observed that this regulatory process was strain-dependent. In infections by the VD strain, increased placental tropism and a robust immune response were detected, potentially reducing the risk of congenital transmission at the expense of cellular metabolism. Maternal parasitemia is another crucial factor associated with congenital risk of transmission.

These findings underscore the notion that congenital transmission of *T. cruzi* is a complex and orchestrated process, wherein various characteristics of both the parasite and the host play pivotal roles in the success of placental infection and subsequent transmission to the fetus. On the other hand, the immune response mechanisms revealed during infection safeguard the placenta from parasitic infiltration, thereby reducing parasitic load and minimizing the risk of congenital infection. Congenital infection has been linked to premature birth, low birth weight, developmental delay, and elevated mortality rates. Moreover, 30% of the infants infected at birth may manifest symptomatic forms in the future. Given the extensive geographical distribution of the parasite and the substantial number of mothers affected by *T. cruzi*, establishing a robust healthcare network is essential. This network should facilitate serological screening of pregnant women, ensuring prompt diagnosis and treatment of newborns. Furthermore, studies that explore the risk markers and prognoses of congenital infections that span diverse geographic regions are necessary. Vertical transmission is not explicitly associated with a particular *T. cruzi* DTU, manifesting through various genetic groups in distinct geographic regions. Most investigations are concentrated in Argentina and Bolivia, mainly focusing on TcV. Expanding the surveillance of congenital transmission and broadening the examination of strains/genetic groups prevalent in other areas is essential to better understand transmission risks and develop intervention proposals.

## 8 Impact of *T. cruzi* DTUs in the Chagas disease treatment

Since the beginning of the 1970s, two drugs have been used for the treatment of *T. cruzi* infection: nifurtimox (NF) (launched by Bayer in 1967, Lampit^®^) and benznidazole (BZ) (launched by Roche in 1972, Rochagan^®^ and Radanil^®^) (306). These nitroheterocyclic compounds require prolonged treatment in patients infected with *T. cruzi* (frequently 60 days) and usually exhibit undesirable adverse reactions that hamper treatment continuity (306, 307). The therapeutic effectiveness of these two drugs varies according to the phase of infection in the patients with Chagas disease (308–310). Numerous studies have reported up to 80% parasitological cure in the acute phase (311) and 60%–70% cure in the recent chronic phase of the disease, in children up to 14 years of age (312–315). However, the major limitation of Chagas disease treatment is its low efficacy in the chronic phase, with only 5%–20% of patients considered cured, as assessed by ≥20 years or more follow-up (306, 310, 316–320). However, benznidazole treatment in the chronic phase can improve the prognosis and clinical outcome in patients with Chagas cardiomyopathy (314, 321–323).

The high variability in the efficacy of benznidazole and NF treatments is controversial, and some studies have attributed it to *T. cruzi* genetic factors. This could explain the differences observed in the screening results when comparing distinct geographic areas (112, 268, 322). The clonal evolution model postulated for *T. cruzi* (266) indicated an association between the phylogenetic divergence of parasite clones and the natural variation in drug susceptibility among *T. cruzi* strains, which is considered an important factor that explains the therapeutic failure in some treated patients with Chagas disease (85, 324–328). Brener et al. (1976) (329) were the first to indicate the heterogeneity of the responses of *T. cruzi* strains to specific treatments. A pioneering study (325) demonstrated a large gradient of drug efficacy for nifurtimox and benznidazole (from 0% to 100%) when 47 *T. cruzi* strains isolated from humans, vectors, and sylvatic reservoirs were investigated in murine models. According to this study, Andrade (1985) (112) observed that during the acute phase, hybrid *T. cruzi* strains were highly susceptible, *T. cruzi* II (TcII) strains were partially to highly susceptible, and *T. cruzi* I (TcI) strains were highly resistant to benznidazole and nifurtimox. Corroborating this study, the authors also verified greater parasitological test negativity in mice infected with Type III (TcI) strains (85.3%) compared to mice infected with Type II (TcII) strains (43%) in the chronic phase after treatment with benznidazole. In animals in the chronic phase, treated with nifurtimox and infected with Type III (TcI) and Type II (TcII) strains, parasitological test negativity was 71.4% and 66%, respectively (330). Similarly, Andrade et al. (1992) (326) demonstrated that in mice treated with benznidazole or benznidazole plus nifurtimox, the cure rate was 66%–100% in those infected with Type II strains (TcII), while in the animals infected with Type III strains (TcI), the cure rate was 0%–9%. The authors indicated that the correlation between the treatment results in patients and mice was 81.8% (9 of 11 cases). Similarly, Filardi and Brener (1987) (324) reported natural resistance to drugs used to treat *T. cruzi* infection in strains or clones isolated from both wild and domestic cycles. *T. cruzi* strains from different geographical areas and isolated from patients with chronic Chagas disease, triatomines, and wild reservoirs were inoculated into mice and showed a gradient of drug susceptibility ranging from 0% to 100%. The correlation between *T. cruzi* strains and the treatment response of Chagas disease was evaluated by Guedes et al. (2002) (331) using dogs as experimental models. The researchers demonstrated that all dogs infected with *T. cruzi* Y strain (TcII) and treated with benznidazole in the acute phase were cured. Moreover, five animals showed antibody levels similar to those of untreated controls, one infected with Berenice-78 (TcII), and four with Colombian strain (TcI), while complement-mediated lysis (CoML) was persistently positive 6 months post-treatment in five dogs, one of which was infected with Berenice-78 (TcII) and four with the Colombian strain (TcI). This study showed that *T. cruzi* genetic variability probably influenced the treatment response, as *T. cruzi* strains from TcI DTU showed more significant resistance to treatment with benznidazole and nifurtimox than *T. cruzi* strains from TcII DTU.

Some authors also consider that *T. cruzi* genetic aspects can explain the differences observed after treatment in distinct geographic areas (7, 315, 332). Yun et al. (2009) (315) showed that in patients with Chagas disease from Honduras, Guatemala, and Bolivia treated with benznidazole, the seroconversion varied widely, even within the same country. Different parasite lineages in distinct geographic regions may explain the differences in seroconversion rates.

Toledo et al. (2003) (85), in a well-designed research, evaluated 20 *T. cruzi* clones isolated from vectors and hosts from different endemic regions and tested the benznidazole and itraconazole susceptibilities during the acute and chronic phases of experimental infection in BALB/c mice. The authors found differences in susceptibility to treatment between the *T. cruzi* stocks according to the genotype, phase at infection, and the drug used in this work. Mice infected with genotype 19 (TcI) or genotype 39 (hybrid) were partially susceptible, while those infected with genotype 20 (TcI) were resistant, and those infected with genotype 32 (TcII) were susceptible to treatment with benznidazole in both infection phases. Considering the treatment with itraconazole drug, mice infected with genotype 19 (TcI), 20 (TcI), or 39 (hybrid) were resistant in the acute phase, and those infected with genotype 32 (TcII) were partially susceptible to treatment. In the chronic phase, animals infected with genotype 19 (TcI) or genotype 20 (TcI) were resistant, those infected with genotype 39 (hybrid) were partially susceptible, and those infected with genotype 32 (TcII) were susceptible to itraconazole treatment. In general, this study demonstrated that clones that belong to TcI DTU were resistant to benznidazole, mainly genotype 20 (TcI), whereas the resistance to itraconazole was observed in both genotypes 20 (TcI) and 19 (TcI). Moreover, genotype 32 (TcII) showed a typical susceptibility profile to benznidazole and itraconazole. Furthermore, Toledo et al. (2004) (333) observed that specific treatment of *T. cruzi* infection altered the parasitological and histopathological parameters according to the parasite genotype (333). Animals infected with genotype 19 (TcI) showed a reduction in tissue parasitism, whereas those infected with genotypes 20 (TcI) and 32 (TcII) showed a decrease in the inflammatory process after benznidazole treatment in the acute phase. In this study, mice infected with genotype 39 (TcV) showed reduced tissue parasitism after treatment with benznidazole in the chronic phase. These data corroborate previous results that indicated that TcI DTU is more resistant to treatment, but in contrast also revealed a positive impact of benznidazole treatment in the pathogenesis of mice infected with this genetic group, even though there is no parasitological cure. The biological parameters of *T. cruzi* clones belonging to different genotypes were also tested in the treatment response to *T. cruzi* infection *in vitro* (99, 334). Revollo et al. (1998) (99) investigated the susceptibility to treatment of *T. cruzi* stocks isolated from various ecological cycles, places, and hosts. They showed that genetic groups 19 (TcI) and 20 (TcI) of the epimastigote and amastigote forms of *T. cruzi* were less sensitive to treatment with benznidazole or nifurtimox. Nonetheless, some studies have encountered challenges establishing a definitive correlation between *T. cruzi* DTU and therapeutic response in Chagas disease. These findings imply that additional factors play an important role in the observed variations in therapeutic efficacy among patients with Chagas disease (335–337). Murta et al. (1998) (335) observed that only strains classified as zymodeme ZB (hybrid) exhibited susceptibility to benznidazole and nifurtimox treatment, whereas zymodemes Z1 (TcI) or Z2 (TcII) displayed a diverse pattern in treatment response, being either susceptible or naturally resistant to these nitroderivatives. Similarly, Moreno et al. (2010) (337) did not find a correlation between *in vitro* drug susceptibility and *T. cruzi*. The authors conducted *in vitro* evaluations of benznidazole sensitivity in *T. cruzi* isolates obtained from seven chronic patients before benznidazole administration and at various intervals after therapy. All isolates were typed as TcII, and three patients (3/7) were considered cured.

Villarreal et al. (2004) (336) investigated potential correlations between *in vitro* benznidazole susceptibility and *T. cruzi* genetic diversity. This study demonstrated that the natural susceptibility of *T. cruzi*, expressed as IC_50_ (concentration achieving a 50% inhibition of parasite growth), was not associated with its genetic structure represented by distinct DTUs. The authors concluded that this result likely stems from the high intra-DTU variation of the surveyed IC_50_ values.

In the context of Chagas disease, the clinical and epidemiological landscapes in the Brazilian Amazon markedly differ from classical endemic transmission areas, possibly influenced by the genetic and biological characteristics of circulating *T. cruzi* DTUs in this region (117). Multiple studies have investigated the susceptibility of *T. cruzi* strains from the Amazon to benznidazole (117, 118, 338). Monteiro et al. (2013) (117) examined *T. cruzi* isolates from the Western Amazon Region that were attributed to TcI and TcIV in Swiss mice. They observed that, although TcI exhibited higher mortality rates in the early chronic phase, a greater frequency of mice with inflammatory processes in any organ, and a higher frequency of mice with tissue parasitism, this DTU showed more susceptibility to benznidazole than TcIV. Teston et al. (2013) (118) investigated the susceptibility of natural *T. cruzi* populations from the state of Amazonas, belonging to TcI and TcIV, in comparison with strains of TcI and TcII DTUs from the states of Paraná and Minas Gerais. Although the researchers found no clear association between susceptibility to benznidazole and *T. cruzi* DTUs, it is demonstrated that TcIV strains exhibited more significant reductions in parasitological, molecular, and serological parameters after treatment than TcI and TcII strains. Notably, TcI strains from Amazonas were significantly more sensitive to benznidazole than TcI strains from Paraná. The extensive genetic variability of TcI isolates from different geographical regions supports the proposition that at least four haplotypes are associated with distinct transmission cycles of the parasite (123, 287, 339, 340). Studies assessing the response to specific chemotherapy in patients from outbreaks related to oral transmission in areas predominantly inhabited by TcI in the Amazon Region demonstrated high parasitological cure rates and decreased antibody titers (341, 342). This evidence supports the notion that TcI isolates from the Brazilian Amazon exhibit dissimilar behavior compared to other regions. The genetic heterogeneity of the TcI suggests that correlations between genetic and biological properties should be investigated at the sub-DTU level. Consistent with the findings of Teston et al. (2013) (118), Gruendling et al. (2015) (338) found no variation in parasitemia, infectivity, or mortality among DTUs when comparing the impact of benznidazole treatment during the acute phase in mice inoculated with TcI and TcIV strains from Amazonas and TcII strains from Paraná, but the proportion of parasitized organs varied with the genetic group.

Disparities in the therapeutic response to Chagas disease treatment have been demonstrated within geographical regions that predominantly harbor the same *T. cruzi* DTU (100, 343). Oliveira-Silva et al. (2015) (343) evaluated the efficacy of benznidazole in both acute and chronic phases in mice infected with TcII isolated from children in the Jequitinhonha Valley, state of Minas Gerais, Brazil, before treatment initiation. This study revealed that most strains resisted benznidazole in both phases, as evidenced by positive parasitological and/or serological tests. Nevertheless, the authors concluded that the treatment yielded benefits, noting a reduction and/or suppression of parasitemia in mice infected with all strains during the acute phase, associated with a reduction/elimination of inflammation and fibrosis. These findings contrast with those of Toledo et al. (2003) (85), who studied clonal stocks of *T. cruzi* with different genotypes, including TcII (equivalent to genotype 32), and reported cure rates of 80% and 69.2% in mice treated during the acute and chronic phases, respectively. Variance in the drug response of *T. cruzi* TcII strains could be attributed to the distinct geographical origins of the parasite populations used in the two studies (343). In another study, Oliveira et al. (2017) (100) assessed the susceptibility of mice to nifurtimox treatment using strains isolated from patients with chronic Chagas disease from the same region of Berilo in the Jequitinhonha Valley. Consistent with the observations of Oliveira-Silva et al. (2015) (343), they noted that only 25% of mice infected with the TcII strain and treated during the acute phase presented negative enzyme-linked immunosorbent assay (ELISA) results in 180 days post-treatment (100). However, the researchers concluded that the treatment had a positive impact, as animals infected with TcII or TcVI exhibited a reduction or elimination of parasitemia and inflammatory lesions.

Various drugs have been tested as alternatives to nitroderivatives against a range of *T. cruzi* strains (344, 345). Molina et al. (2000) (344) investigated the *in vivo* activity of posaconazole against a series of *T. cruzi* strains in both immunocompetent and immunosuppressed murine hosts. The results of this study revealed that in mice infected with the CL strain (TcVI), posaconazole at 20 mg/kg/day successfully cured 100% of the surviving animals, with a cure rate comparable to that achieved with benznidazole at 100 mg/kg/day. In contrast, mice infected with the Y strain (TcII) exhibited a cure rate of less than 50% with 100 mg/kg/day benznidazole, whereas the cure rate for animals receiving 20 mg/kg/day posaconazole approached 90%. Notably, in mice infected with the Colombian strain (TcI), the benznidazole at 100 mg/kg/day failed to induce a parasitological cure. However, 50% of the surviving animals that received posaconazole at a dose of 20 mg/kg/day were successfully cured. The authors suggested that posaconazole was effective against both susceptible and drug-resistant parasite populations in murine hosts. To ascertain the effectiveness of current and prospective drugs against diverse *T. cruzi* stocks, Moraes et al. (2014) (345) evaluated the *in vitro* activities of different compounds against six *T. cruzi* DTUs. Major differences in the susceptibility or resistance of strains and clones to the tested drugs were not found, indicating that DTUs may not directly correlate with a specific medication resistance pattern *in vitro*.

Additionally, ergosterol biosynthesis inhibitors have been introduced as new chemotherapeutic agents for treating Chagas disease. Triazoles such as posaconazole and ravuconazole, essential for ergosterol biosynthesis, have demonstrated potent trypanocidal activity both *in vitro* and *in vivo* (201, 344, 346–350). Among the most advanced and promising compounds is another nitroheterocyclic compound, fexinidazole, which exhibits activity against *T. cruzi* (351). Parallel efforts led to the discovery of the oxaborole class, specifically AN4169 (SCYX-6759), which demonstrated curative antichagasic activity in a mouse model and is now positioned as an innovative drug for the treatment of Chagas disease (352).

Collectively, these studies demonstrate that the genetic variability of *T. cruzi* alone is insufficient to account for the variations observed in treatment responses comprehensively. An essential consideration in Chagas disease is the occurrence of mixed infections in vertebrate and invertebrate hosts (353, 354). Substantial interactions within the subpopulations of these mixtures have been observed, leading to alterations in the biological properties of the parasite (94, 104, 105, 355). Such changes can potentially influence morbidity, the dynamics of *T. cruzi* transmission, and the response to chemotherapy in Chagas disease. Martins et al. (2007) (95) investigated the consequences of dual infections with clonal stocks of *T. cruzi* genotypes on the efficacy of benznidazole treatment in a murine model. Their investigation revealed that mixed infections exhibited treatment responses distinct from single-infection analyses’ predictions. The authors verified that 9 out of 24 dual infections shifted the predicted benznidazole susceptibility profile compared to the respective single infections. Notably, six dual infections involving almost all tested genotype combinations demonstrated an increase in the cure rate, except for 19 + 32 (TcI + TcII) and 20 + 19 (TcI + TcI), while two exhibited a reduction, including genotypes 19 + 39 (TcI + TcV) and 19 + 32 (TcI + TcII). The researchers concluded that mixed *T. cruzi* infections, a phenomenon observed in nature, could significantly impact the efficacy of chemotherapy for Chagas disease.

Some studies suggest that additional factors should be considered when assessing the response to Chagas disease treatment (99, 334). Different life stages of the parasite can influence the susceptibility of various *T. cruzi* strains to benznidazole and nifurtimox (334, 356, 357). Canavaci et al. (2010) (356) demonstrated that the *in vitro* susceptibility of intracellular amastigotes of CL and Colombian strains is indistinguishable even though these strains are well known for their susceptibility and high resistance to benznidazole and nifurtimox *in vivo*, respectively (324, 344). Revollo et al. (2019) (334) explored the *in vitro* susceptibility of 21 *T. cruzi* strains belonging to the TcI, TcII, and TcV DTUs to epimastigotes, trypomastigotes, and amastigotes. These strains were isolated from patients, reservoirs, and triatomine bugs with distinct geographic origins. The study employed a methodology based on computing the epidemiological cutoff value (COwt) of *T. cruzi* stages against benznidazole and nifurtimox. Using a panel of previously characterized strains, the researchers examined the frequency of the sensitive (wild-type) phenotype within the three DTUs. The findings revealed that epimastigotes are more susceptible to both drugs than intracellular amastigotes, whereas trypomastigotes globally exhibit a higher inherent capacity to resist the effects of both benznidazole and nifurtimox. Moreover, regardless of the stage studied, strains belonging to TcI were more frequently identified as less susceptible to treatment than TcII and TcV. The drug susceptibility of *T. cruzi* is related to the genotype but is also linked to the life history trait of each strain (334).

Recently, a stage in the intracellular life cycle of *T. cruzi*, referred to as dormant amastigotes (258, 358), has been identified, which appears to influence the response to treatment. In addition to variations in their replicative capacity, these dormant amastigotes exhibit differences in their transcriptomes and more significant resistance to treatment during their non-replicative stages (358, 359). The authors demonstrated that this phenotype is transient, and dormant amastigotes revert to actively replicating forms susceptible to the drug. Consequently, trypanocidal drugs efficiently eliminate actively replicating amastigotes but not dormant forms, suggesting that dormancy is the primary factor contributing to the ineffectiveness of these drugs in achieving a consistent parasitological cure (360). Therefore, a treatment protocol combining high doses, reduced frequency, and extended treatment duration would likely be more effective in addressing and eliminating dormant parasites (360) and achieving therapeutic success.

Indeed, the response to treatment for Chagas disease is understood to be a multifactorial and intricate process with numerous gaps yet to be addressed. Therapeutic failure encompasses a set of factors associated with the host (including genetic and immunological traits), infective agent (genetics and acquired drug resistance), drug used (pharmacodynamics/pharmacokinetics), and chemotherapeutic protocol (334). Differences in assay methodology, including the life cycle stage of the parasite used in the test and variation in *T. cruzi* strain population composition among different laboratories, should be duly considered (99, 334, 345). Additionally, it is crucial to compare the *in vitro* and *in vivo* assays. Given that most reports are based on mouse models, it is impossible to definitively attribute therapeutic failure to “natural” intrinsic *T. cruzi* resistance (345). *In vivo* chemotherapy involves several parameters that are often uncontrolled even in experimental models, such as the relationship between exposure and cure, route of administration, time of treatment, treatment regimen and length, mouse lineage, and type of immune response, along with other Chagas disease-related challenges, such as definitions of cure criteria and disease stage (345). It is also essential to consider the lesser phylogenetic subdivisions because of the heterogeneous behavior of strains within the same broad genetic subdivisions (85, 95). The biological characteristics of the parasite, such as susceptibility to treatment, may vary even within the same *T. cruzi* DTU (100, 343, 361). Because of the myriad factors that can impact the therapeutic response to Chagas disease, a priority in scientific research should be the development of new drugs with improved efficacy, shorter treatment course, fewer side effects, and resilience against *T. cruzi* genetic variability and dormant forms.

## 9 Methods used for the genotype-specific diagnosis of Chagas disease

The methodologies used for the genotype-specific diagnosis of Chagas disease to recognize *T. cruzi* DTUs have been developed, including biochemical and molecular methods (5, 7) as well as serological tests (362–371). Considering the distinct geographic distribution of DTUs (7), genotype-specific diagnosis of Chagas disease is relevant for implementing epidemiological surveillance and more precise strategies for disease control in endemic areas. Moreover, genotyping methods can also be helpful in establishing the clinical prognosis status of ongoing infection since different *T. cruzi* DTUs have been associated with distinct severity of Chagas disease (20, 72, 372). In addition, considering the divergent susceptibility of *T. cruzi* DTUs to the etiological treatment of Chagas disease (20, 85, 95), the methodologies used for the genotype-specific diagnosis of *T. cruzi* infection are important for post-therapeutic monitoring of treated patients.

*T. cruzi* genetic variability may interfere with the sensitivity of the methods used for Chagas disease diagnosis, mainly in serological tests. Previous studies have shown that patients from different geographic areas infected with distinct *T. cruzi* DTUs seem to display differential serological patterns upon diagnosis of Chagas disease (373–376). Verani et al. (2009) (375) verified two serological tests using serum samples from Bolívia and Peru, with distinct sensitivities ranging from 26.6% to 87.5%, corroborating the hypothesis that the intrinsic features of regional *T. cruzi* strains can impact serological tests. Thus, considering the genetic variability of *T. cruzi*, it is important that the antigens for the universal diagnosis of Chagas disease are expressed in different DTUs (376).

Currently, molecular methods are broadly used for genotype-specific diagnosis of single and mixed *T. cruzi* infections during the acute and chronic phases (5). Classification of *T. cruzi* in genetic groups was based on a variety of molecular targets by several techniques such as polymorphism of rDNA and mini-exon (377, 378), multilocus enzyme electrophoresis (MLEE) (4, 21, 379), multilocus sequence typing (MLST) (47, 380–382), restriction fragment length polymorphism (PCR-RFLP) (11, 276, 383, 384), random amplified polymorphic DNA (RAPD) (385), microsatellite typing (MLMT) (340, 386–388), target-specific PCR (22, 61, 287), PCR-DNA blotting with hybridization assays (389–392), and amplicon deep sequencing (290).

The method of Lewis et al. (2009) (6) has been commonly used for *T. cruzi* genotyping. This method is based, in principle, on the analysis of the divergent domain D7 of 24Sα subunit rDNA (LSU rDNA) (16) and two restriction fragment length polymorphisms (PCR-RFLP) of HSP60 (heat shock protein) and GPI (glucose 6-phosphate isomerase) genes. Combining these three methodologies enables the typing of most *T. cruzi* strains with good reproducibility (6). Another method broadly used for *T. cruzi* genotyping was proposed by D´Ávilla et al. (2009) (228), which is based on the PCR-RFLP of the subunit II of the mitochondrial enzyme cytochrome oxidase (COII) gene region and the amplification of the intergenic region of the spliced leader (SL-IR) of the miniexon gene (276), as well as the analyses of the divergent domain D7 of 24Sα subunit rDNA (LSU rDNA). This criterion also showed good reproducibility and identified mixed infections with different *T. cruzi* genotypes (228). De Oliveira et al. (2015) (67) identified the genetic profile of 63 *T. cruzi* samples isolated from patients with chronic Chagas disease in two municipalities of Jequitinhonha Valley, MG, Brazil, using protocols proposed by Lewis et al. (2009) (6) and D´Ávilla et al. (2009) (228). The authors demonstrated that the criteria of Lewis et al. (2009) (6) identified 89% of the samples as TcII, but it was not possible to define the genetic identity of seven isolates, and the criteria proposed by D´Ávilla et al. (2009) (228) corroborated the classification as TcII for the same samples and defined the classification of the other seven as TcVI. Presently, the methods used for typing *T. cruzi* DTUs are based on polymorphism of the mini-exon gene (Spliced Leader) and the 24Sα and 18S ribosomal RNA (22). Multilocus PCR-RFLP (MLP), which analyzes the genetic polymorphism of different *T. cruzi* loci (27), MLEE (379), and RAPD (21) employing different markers and steps have also been used for parasite genotyping mainly in single *T. cruzi* infections. Other assays include the analysis of complex electrophoretic patterns generated by restriction polymorphisms in PCR-amplified genomic DNA (384, 393, 394). These methods are able to discriminate *T. cruzi* DTUs, but they are work- and time-consuming, and the interpretation of the results may be misleading. Some molecular methods can be used for *T. cruzi* genotyping directly from biological samples, such as the unique low astringency specific PCR primer technique (LSSP-PCR) (125, 212) by analysis of the rDNA polymorphism (276, 395) and examination of the microsatellite polymorphism (263, 396), which are important for the best understanding of the pathogenesis of Chagas disease.

Real-time quantitative PCR (qPCR) is able to amplify satellite DNA (SatDNA) sequences from the *T. cruzi* genome (397–399). The qPCR detects variations in the SatDNA sequences of different *T. cruzi* genetic groups; therefore, this variability must be considered when estimating the parasitic load. This technique showed different sensitivities between the different DTUs of the parasite: TcI < TcII < TcV and TcVI (398). Cura et al. (2015) (400) used the real-time PCR with TaqMan probes (MTq-PCR) to identify *T. cruzi* mixed infections with parasite isolates from humans, triatomines, and wild reservoirs. The Multiplex Real-Time PCR assay using TaqMan probes was useful for determining the *T. cruzi* DTUs of cultures, vectors, and blood samples from patients in the acute phase (384, 394). Muñoz-San et al. (2017) (401) used the real-time PCR for genotyping of 53 blood samples from individuals with chronic Chagas disease, and revealed that the method was able to characterize 67.9% of samples, with high detection of TcII, followed by TcI. Muñoz-San et al. (2018) (402) also genotyped 86 samples of patients with chronic Chagas disease from Chile by real-time PCR assay and determined whether *T. cruzi* DTUs are related to the presence of heart disease. The data obtained in this study showed that in the group with cardiomyopathy, the most frequent DTU was TcI (56.1%), followed by TcII (19.5%), while mixed infections of TcI and TcII were observed in 7.3% of the patients. In this study, in the group without cardiac pathologies, TcI and TcII were found at similar rates (28.9% and 31.1%, respectively), and mixed infections TcI + TcII were found in 17.8% of the cases. Ramírez et al. (2017) (403) analyzed 335 distinct *T. cruzi* SatDNA sequences from 19 *T. cruzi* stocks representative of TcI-TcVI DTUs by real-time PCR for phylogenetic inference. The authors revealed that all sequences were grouped into three major clusters, which corresponded to sequences from TcI/III, TcII, and TcIV, whereas the TcV and TcVI stocks had two sets of sequences distributed into TcI/III and TcII clusters. The major limitation of this approach used in this work is that it cannot distinguish between the presence of hybrid lineages TcV and TcVI and *T. cruzi* mixed infections with TcI or TcIII and TcII strains. Moreover, this study opens the possibility of typing samples from patients with Chagas disease with low parasitic loads and improves molecular diagnostic methods of *T. cruzi* infection based on SatDNA sequence amplification. This genotyping strategy could be useful for patients with chronic Chagas disease with low parasitic loads, whose samples are usually difficult to genotype (400, 404).

Increasingly, DNA markers to *T. cruzi* genotype with high copy numbers, such as kinetoplast minicircles, are needed because of the low number of parasites in biological samples, particularly in patients with chronic Chagas disease. Bontempi et al. (2016) (405) developed a DNA assay called minicircle lineage specific-PCR (MLS-PCR) to genotype reference strains of *T. cruzi* and parasite DTUs isolated directly from biological samples. The researchers verified the high specificity and sensitivity of the new approach for TcI, TcII, TcV, and TcVI genotyping in 22 *T. cruzi* reference strains. Moreover, the MLS-PCR technique and hybridization tests using specific kDNA probes could genotype 94% of the blood samples from patients with chronic Chagas disease in northeastern Argentina. Since there is frequently a low number of parasites in infected tissues or blood samples, genetic markers with a high number of copies are necessary to achieve good sensitivity of detection (406). Rusman et al. (2019) (407) performed deep amplicon sequencing of the hypervariable kDNA minicircles’ hypervariable region in nine strains of six *T. cruzi* DTUs. This work demonstrated that the number and diversity of mHVR clusters were variable among DTUs, and even within a DTU, *T. cruzi* strains of the same DTU shared more mHVR clusters than strains of different DTUs. The authors concluded that mHVR amplicon sequencing is a reproducible technique that allows multiplexed analysis of hundreds of strains by direct genotyping on biological samples, but has the disadvantage of being technically cumbersome, relying on visual interpretation of bands, and requiring representative strains of the diversity of *T. cruzi* in every assay (used as probes). Da Cruz Moreira and Ramirez (2019) (408) amplified several targets from the *T. cruzi* genome, such as SL-IRac, SL-IR I-II, 24Sα rDNA, and A10, by multilocus conventional PCR to genotype the DTUs directly from the blood and other clinical samples, without the need to isolate the parasite before DNA extraction, even at a lower parasite concentration.

One of the main challenges in genotyping biological samples is the identification of mixed infections by different *T. cruzi* DTUs (72). Some methods employing molecular biology can detect mixed *T. cruzi* infections, such as those used in the protocol proposed by D´Ávilla et al. (2009) (228) (114, 409). Andrade et al. (2011) (114) used the PCR-RFLP technique of the subunit II cytochrome oxidase gene and analyzed the polymorphism of the 24sα-LSU rDNA gene, as well as the polymorphism of mini-exon SL-IL intergenic spacer, to demonstrate mixed infections with TcI + TcIII and TcII + TcVI DTUs of *T. cruzi* in parasites isolated from patients with acute Chagas disease, triatomines, and wild reservoirs. Sá et al. (2016) (409) also verified mixed *T. cruzi* infections in the intestinal content of *Rhodinus prolixus* using the criteria of D´Ávilla et al. (2009) (228). The researchers identified 79.4% of mixed *T. cruzi* infections; however, PCR-RFLP of the subunit II cytochrome oxidase gene was not able to recognize mixed infections by TcV+ TcVI, requiring association of this technique with the analyses of the 24sα-LSU rDNA gene polymorphism. Previous studies have shown mixed *T. cruzi* infections by kDNA PCR amplification and subsequent hybridization using DTU-specific probes (DTU-blot) (354, 410), PCR-Real Time with Taq-Man probes (MTq-PCR) (400), and the amplification of other parasite genome targets, such as the cSC5D gene by PCR-RFL (384). Moreover, some studies revealed that parasite populations in blood hosts were distinct from those isolated by xenodiagnosis and grown in culture media (353, 354, 410). Ortiz et al. (2015) (410) demonstrated mixtures of TcI, TcII, TcV, and TcVI in blood samples of patients with chronic Chagas disease via kDNA PCR amplification and subsequent hybridization with DTU-specific probe, while in the xenodiagnosis followed by axenic culture, most parasites were identified with TcV. Even when using different targets from the *T. cruzi* genome, studies such as Consentino and Aguero (2012) (384) and Cura et al. (2015) (400) did not detect all mixed parasite infections in biological samples. Consentino and Aguero (2012) (384), through the PCR-RFLP of the cSC5D gene, did not identify mixed infections from the TcV + TcVI *T. cruzi* DTUs. Cura et al. (2015) (400) were unable to verify the mixed infection from TcI + TcII/TcV/TcVI DTUs in patients with Chagas disease by the PCR-Real Time with Taq-Man probes (MTq-PCR) and failed to detect mixed *T. cruzi* infections in triatomine and wild reservoir samples. Nevertheless, even using high-complexity methodologies, some approaches could not detect all the mixed infections in hosts infected with *T. cruzi*.

The molecular methods used for the genotype-specific diagnosis of Chagas disease have advantages and drawbacks, such as high complexity, extended analysis time, and misleading interpretation of the results, and all of them are based on genetic signatures (411). The genotyping of *T. cruzi* infections in chagasic chronic hosts by these methodologies still represents a challenge, as some of these methods require several genetic markers to distinguish *T. cruzi* genotypes (6, 22, 67, 228, 276). Furthermore, the majority of biochemical/molecular approaches cannot be directly performed in biological and clinical samples, requiring previous parasite isolation by low-sensitivity methods (hemoculture or xenodiagnosis) followed by *in vitro* growth, which may lead to clonal selection (61, 125, 220, 410, 412–414). In addition, according to the Clonal Histiotropic Model, parasite isolates from peripheral blood samples do not necessarily represent the complete set of *T. cruzi* DTUs found in distinct host tissues (126, 127, 221, 234). Parasitemia in patients and reservoirs infected with *T. cruzi* is variable, and the success of parasite isolation is dependent on host parasitemia; therefore, several strategies proposed to genotype *T. cruzi* isolates have been applied only to cultured stocks and biological or clinical samples with high parasitic loads (47, 118, 276, 343, 384, 400, 415, 416). Another limitation is that target sequences of *T. cruzi* amplified by molecular methods for DTU identification are found in a single copy number or low copy numbers; therefore, the use of targets with high copy numbers, such as SatDNA and kDNA mHVR sequences, has a significant impact in clinical and epidemiological studies (69, 70, 403, 407). Given these limitations, further research is required to optimize the sensitivity and simplify methods for genotyping *T. cruzi* DTUs that can be easily applied in clinical laboratories (407, 417).

Previous studies have shown the influence of *T. cruzi* genotype on the antibody response pattern in experimental models (88, 418, 419). The profile of lytic antibodies varies when distinct *T. cruzi* strains are used as target antigens, suggesting that genotype-specific antigenic features of the parasite could be involved in the induction of lytic antibodies (418–420). Dos Santos et al. (2009) (88) correlated *T. cruzi* genetic diversity with the pattern of humoral immune responses in mice infected with different parasite genotypes.

Several innovative serological methods have been developed to overcome the limitations of molecular tests for genotype-specific diagnosis of Chagas disease, mainly during the chronic phase (362–371, 421). The first serological marker to identify *T. cruzi* genotypes was the mucin trypomastigote small surface antigen (TSSA), a surface protein of the bloodstream trypomastigote, which initially appeared to correlate with the parasite lineages *T. cruzi* I (TcI) and *T. cruzi* II (TcII) (422). Bhattacharyya et al. (2010, 2014, 2015) (362, 364, 365) verified that TSSA pepII/V/VI isoforms could distinguish samples of hosts infected with different *T. cruzi* genotypes. Bhattacharyya et al. (2010) (362) identified TSSA II as an antigen capable of recognizing TcII, TcV, and TcVI *T. cruzi* DTUs as distinct from infections by TcI, TcIII, and TcIV DTUs; already the peptide described specific for TcI infections (TSSA I) share some characteristics with as TcIII and TcIV DTUs. The researchers found greater *T. cruzi* lineage-specific diversity than that previously described by Di Noia et al. (2002) (422). Bhattacharyya et al. (2014) (364) evaluated the reactivity of serum samples from infected mice with different *T. cruzi* genotypes and human samples from distinct geographical regions of Latin America by ELISA using the TSSA antigen. The results obtained in this study showed that sera from mice infected with TcII, TcV, or TcVI DTUs recognized the TSSApep-II/V/VI antigen, whereas sera from mice infected with TcIII or TcIV DTUs reacted with their respective TSSA peptides. In this work, the sera of patients with Chagas disease from the Southern Cone reacted with TSSApep-II/V/VI and TSSApep-V/VI antigens; however, the samples of patients with Chagas disease from the Amazon North region did not specifically identify the TcI antigen. Bhattacharyya et al. (2010, 2014, 2015) (362, 364, 365) indicated that the protein core of TSSA contains remarkable polymorphism, in which TcI, TcIII, and TcIV have their lineage-specific sequences; TcII, TcV, and TcVI share a common epitope, and the hybrid lineages TcV and TcVI share an additional epitope. In this context, the authors proposed that TSSA isoforms are feasible serological markers for identifying *T. cruzi* DTUs in humans and experimental infections. Nevertheless, TSSApep-I did not show significant reactivity with human chagasic sera from TcI-endemic regions, potentially due to low immunogenicity or a conformation of the peptide differing from that of the native antigen, suggesting that novel targets for TcI are still required (362, 364, 365). Ker et al. (2016) (366) applied ELISA using lineage-specific TSSA epitopes to the sera of Brazilian primates from the Atlantic Forest and Amazon regions to identify *T. cruzi* DTUs that infect hosts and further expand the knowledge of transmission cycles in these biomes. This study demonstrated that both peptides, TSSApep-II/V/VI and TSSApep-V/VI, gave lineage-specific reactions with samples from the Atlantic Forest; conversely, only one Amazonian primate produced a peptide reaction with only TSSApep-III and TSSApep-IV. The authors concluded that this novel methodology readily applies to many mammalian species. However, for the discovery of *T. cruzi* genotype, more attempts should be made to design an efficacious TcI-specific peptide. Bhattacharyya et al. (2018) (369) developed a rapid diagnostic test (RDT; Chagas Sero K-SeT) that incorporates a peptide that corresponds to the TSSA II/V/VI common epitope and tested it with sera of patients with Chagas disease from Bolivia and Peru, including individuals with varying cardiac pathology, in addition to matched mothers and neonates. This study showed that Chagas Sero K-SeT RDT can replace ELISA for TSSApep-II/V/VI serology and that the response to this RDT was associated with the severity of Chagas cardiomyopathy in Bolivian patients and thus may have prognostic value. Chagas Sero K-SeT RDT using the TSSApep-II/V/VI common epitope peptide and TSSApep ELISA were applied for *T. cruzi* lineage-specific diagnosis in serum samples from humans and mammals in the Chaco region of northern Argentina to gain further insight into ecological and epidemiological associations (423). Chagas Sero K-SeT RDT detected human and canine IgG and was as sensitive as TSSApep-II/V/VI ELISA. Moreover, seropositivity by both methods was 69.5% for humans, 65.8% for dogs, and 14.3% for armadillos; by ELISA, it was 26.3% for cats (423). Another study used the Chagas Sero K-SeT RDT, which incorporates the TSSApep-II/V/VI peptide epitope for *T. cruzi* lineage-specific diagnosis in serum samples from experimentally infected murine and sera from a range of mammalian orders and biomes in Brazil for epidemiological surveillance (421). This method identified the TcII/V/VI genotypes in experimental murine *T. cruzi* infections and natural infections across several orders of Brazilian mammals, namely, Primata, Carnivora (canine), Rodentia, and Chiroptera; however, Chagas Sero K-SeT RDT could not recognize IgG from felines and armadillos (421). The Chagas Sero K-SeT RDT is directly applicable to TcII/V/VI-specific serological surveillance of *T. cruzi* infection among potential animal reservoirs of the parasite, to assess the risk of emergent endemic regions and to guide control strategies without the need to isolate *T. cruzi* from the animals (421, 423). Other *T. cruzi* target antigens have been investigated by Mendes et al. (2013) (363) for application in the ELISA test for genotype-specific diagnosis of Chagas disease. Mendes et al. (2013) (363) identified three polymorphic linear B-cell epitopes on proteins derived from a pair of alleles of the hybrid CL Brener strain genome that could be used for serotyping Chagas disease by ELISA. These authors tested serum samples from mice infected with Colombian (TcI), CL (TcVI), and Y (TcII), as well as sera from patients with Chagas disease infected with TcI and TcII DTUs. This study demonstrated that three polymorphic epitopes could discriminate TcI and TcII infections; however, samples from animals infected with TcVI showed cross-reactivity with the peptides. This study demonstrated the potential of these target antigens for Chagas disease serotyping, but it is necessary to improve antigen search and develop a robust panel of strain-specific epitopes to achieve a method applicable to large epidemiological studies.

Previous studies have developed a new serological method for improving genotype-specific diagnosis of Chagas disease to provide the profile of *T. cruzi* DTUs that infect the patients (367–369). A flow cytometry-based technique, called Chagas-Flow ATE (Amastigote, Trypomastigote, and Epimastigote), was developed to assess the expression of anti-amastigote, anti-trypomastigote, and anti-epimastigote antibodies, displaying high performance for the diagnosis and post-therapeutic monitoring of Chagas disease (357). Subsequently, Alessio et al. (2017) (367) optimized the Chagas-Flow ATE for genotype-specific diagnosis of a single experimental *T. cruzi* infection in the chronic phase, using as target antigens parallel batches of distinct *T. cruzi* genotypes representative of three major DTUs involved mainly in the domestic (TcI, TcII, and TcVI) and sylvatic (TcI) cycles of Chagas disease in Brazil (4, 5). The researchers demonstrated that the Chagas-Flow ATE methodology showed 74% global accuracy in discriminating infections with TcI vs. TcVI vs. TcII DTUs and 94% global accuracy in segregating infections with TcI vs. TcII, showing its applicability for genotype-specific diagnosis of single experimental *T. cruzi* infection in the chronic phase. To extend the early studies carried out by Alessio et al. (2017) (367), the same research group evaluated the performance of the Chagas-Flow ATE methodology for early and late differential diagnosis of single and dual genotype-specific experimental *T. cruzi* infections (368). Alessio et al. (2018) (368) revealed that during the early stage, this new method presented 72% global accuracy in differentiating single infections and 80% global accuracy in segregating *T. cruzi* dual infections, while in the late stage, the global accuracy was 69% for discriminating single infections and 76% for *T. cruzi* dual infections. The authors concluded that Chagas-Flow ATE showed good performance for genotype-specific diagnosis of single and dual experimental *T. cruzi* infections, in both early and late stages, indicating that this technique presented an accuracy of 81% as a complementary diagnostic test for *T. cruzi* infection (368). Alessio et al. (2020) (371) evaluated the performance of Chagas-Flow ATE for DTU-specific diagnosis of Chagas disease using serum samples from patients with Chagas disease chronically infected with TcI, TcVI, or TcII DTUs of *T. cruzi.* The results obtained in this work demonstrated the high performance of the Chagas-Flow ATE technique for DTU-specific diagnosis of Chagas disease, with 92% global accuracy in discriminating “TcI vs. TcVI vs. TcII” and 97% global accuracy in segregating “TcI vs. TcII” *T. cruzi* infections. Earlier studies were pioneers in the development of a serological method by flow cytometry for the genotype-specific diagnosis of Chagas disease, with advantages such as the use of a wide range of antigenic preparations in a single flow cytometric platform (357, 424–427), testing of a large number of samples, and evaluation of the reactivity of serum samples from single and mixed *T. cruzi* infections (367, 368, 371).

Innovative work proposed a method based on mass spectrometry for genotyping *T. cruzi* DTUs (411). *T. cruzi* revealed differential protein expression in distinct genetic groups of parasites (411). De Oliveira et al. (2018) (411) developed a *T. cruzi* Strain Typing Assay using MS2 peptide spectral libraries (Tc-STAMS2). The method was based on constructing a spectra library, which was inspected using MS/MS spectra from unknown *T. cruzi* strains and assigned to a specific strain in an automated and computationally driven approach. Therefore, using shotgun proteomics combined with spectral library search, *T. cruzi* strains can be discriminated independently from genome knowledge. The Tc-STAMS2 method identified more than 4,000 proteins in the combined six DTUs strains, and 1,096 proteins were differentially expressed between the six DTUs; multivariate analysis allowed the discrimination of *T. cruzi* strains using the quantitative MS signal. The authors concluded that Tc-STAMS2 represents a complementary strategy for genotyping *T. cruzi* DTUs using only fragmentation spectra without needing genomic data (411).

The identification of new biomarkers for use in *T. cruzi* genotyping is a current research challenge (417). A primary focus in Chagas disease studies is establishing an association between DTUs and clinical manifestations of the disease or response to treatment (417). Therefore, methods of discerning single and mixed *T. cruzi* infections during diagnosis are paramount. This capability directly impacts predicting disease morbidity, assessing the response to chemotherapy, and anticipating reactivation in immunocompromised patients (65, 402, 428). Furthermore, the notable genetic diversity of *T. cruzi*, coupled with the distinct geographic distribution and transmission cycles of DTUs, elevates the significance of their identification. This aspect is crucial for ecological and epidemiological surveillance, laying the groundwork for precise disease control strategies (5, 7, 20).

## 10 Discussion

The clinical outcome of Chagas disease is influenced by several factors, among which the most important are the host’s immune system and the biological and genetic characteristics of the parasite. Several studies have demonstrated that the genetic variability of *T. cruzi* directly influences its infectivity and virulence. These factors, which are closely linked to the epidemiology and immunopathogenesis of the disease, play an important role in the response of infected individuals and the evolution of Chagas disease. This study aimed to describe some aspects of *T. cruzi* genetic diversity, its interaction with the host, and the principal methodologies used for genotyping the parasite. In this context, several methodologies are available for genotype-specific diagnosis of Chagas disease, including molecular methods. Each of these approaches has its advantages and drawbacks, so the choice of which should be used in *T. cruzi* genotyping must take into account aspects such as the type of sample, the presence of mixed parasite infections, the genetic marker, and the process of DNA or RNA extraction (in the molecular methods). The geographic distribution of the different DTUs of *T. cruzi* can influence the parasite’s transmission cycle, the host’s adaptability, treatment response, and the infected individuals’ clinical course.

The genetic variability of *T. cruzi* plays a crucial role in parasite infectivity, reproduction, and differentiation within vectors. Some strains may exhibit distinct distributions among hosts, signifying preferential tissue tropism, particularly in *T. cruzi* mixed infections. Conversely, studies indicating a lack of correlation between the clinical manifestations of Chagas disease and *T. cruzi* DTUs should be considered. The genetic makeup of *T. cruzi* is directly associated with disease reactivation events and symptoms in immunosuppressed patients. Although genetic variability significantly contributes to the pathogenesis of the disease, further investigations are imperative to comprehensively assess these aspects, particularly within the context of mixed infections and reinfections. In this milieu, strains comprising different *T. cruzi* DTUs and distinct populations involved in infection have the potential to modulate the immune response, thereby influencing the pathogenesis of the disease and response to treatment. Evaluation of intragroup variability and parasite gene expression should be conducted more comprehensively to enhance our understanding of the impact of this parameter.

It is currently difficult to determine a direct association between the genetics of *T. cruzi* and the development of different clinical conditions in Chagas disease. However, identifying the different immunological and pathogenic mechanisms triggered by different strains can provide important information for better monitoring affected patients and directing possible therapies. Knowledge of the genetic variability of *T. cruzi* and its countless implications in the course of disease in infected individuals may contribute to strategies for control, clinical prognosis status of ongoing infection, and post-therapeutic monitoring of the disease. Thus, it is necessary to carry out studies that address in an integrated manner all aspects related to the genetic variability of *T. cruzi* and their implications in the different outcomes of Chagas disease.

## Author contributions

MM: Writing – review & editing, Writing – original draft, Methodology, Investigation, Conceptualization. GA: Writing – review & editing, Writing – original draft, Methodology, Investigation, Conceptualization. BF: Writing – original draft, Methodology, Investigation, Conceptualization. PS: Writing – review & editing, Methodology, Investigation, Data curation, Conceptualization. MA: Writing – review & editing, Methodology, Investigation, Data curation, Conceptualization. CS: Writing – review & editing, Methodology. GB: Writing – review & editing, Methodology, Investigation, Data curation, Conceptualization. OM-F: Writing – review & editing, Supervision, Methodology, Investigation, Funding acquisition, Data curation, Conceptualization. AT-C: Writing – review & editing, Supervision, Project administration, Methodology, Investigation, Funding acquisition, Data curation, Conceptualization. HM: Writing – review & editing, Writing – original draft, Supervision, Project administration, Methodology, Investigation, Funding acquisition, Data curation, Conceptualization.
